# Odorant receptors can mediate axonal identity and gene choice via cAMP-independent mechanisms

**DOI:** 10.1098/rsob.160018

**Published:** 2016-07-27

**Authors:** Kiavash Movahedi, Xavier Grosmaitre, Paul Feinstein

**Affiliations:** 1 Max Planck Institute of Biophysics, Max-von-Laue-Strasse 3, 60438 Frankfurt, Germany; 2 Myeloid Cell Immunology Laboratory, VIB Inflammation Research Center, Ghent, Belgium; 3 Laboratory of Cellular and Molecular Immunology, Vrije Universiteit Brussel, Brussels, Belgium; 4 Centre des Sciences du Goût et de l'Alimentation, CNRS, INRA, Université Bourgogne Franche-Comté, 21000 Dijon, France; 5 Department of Biological Sciences, Hunter College and The Graduate Center Biochemistry, Biology and Biopsychology and Behavioral Neuroscience Programs, CUNY, New York, NY, USA

**Keywords:** olfactory receptor, axon guidance, olfaction, gene choice, GPCR, neuronal wiring

## Abstract

Odorant receptors (ORs) control several aspects of cell fate in olfactory sensory neurons (OSNs), including singular gene choice and axonal identity. The mechanisms of OR-induced axon guidance have been suggested to principally rely on G-protein signalling. Here, we report that for a subset of OSNs, deleting G proteins or altering their levels of signalling does not affect axonal identity. Signalling-deficient ORs or surrogate receptors that are unable to couple to Gs/Golf still provide axons with distinct identities and the anterior–posterior targeting of axons does not correlate with the levels of cAMP produced by genetic modifications. In addition, we refine the models of negative feedback by showing that ectopic ORs can be robustly expressed without suppressing endogenous gene choice. In conclusion, our results uncover a new feature of ORs, showing that they can instruct axonal identity and regulate olfactory map formation independent of canonical G-protein signalling and cAMP production.

## 1. Introduction

Olfaction detects chemosensory stimuli with an enormous diversity in physico-chemical properties. To accommodate this broad recognition, the olfactory system employs a large repertoire of odorant receptors (ORs) [1,2]. OR genes form the largest multi-gene family in mammals, with the mouse having approximately 1100 functional receptors and approximately 200 pseudogenes [3]. Signal transduction through the OR is derived by ligand binding (odour) catalysing the bound heterotrimeric G-protein complex to exchange GDP for GTP in the Gα subunit and the dissociation from the Gβγ dimer. Gα bound to GTP subsequently activates adenylyl cyclase 3 (Adcy3), inducing the production of cyclic AMP (cAMP), which then binds to cyclic nucleotide-gated ion channels and depolarizes the cell membrane [4]. While one would expect that the OR's main role would be to detect odorants and initiate signal transduction, extensive evidence has shown that ORs also play pivotal roles in the development of the olfactory system [5,6]. However, dissecting the pluralistic roles of ORs has been challenging and their functions remain enigmatic.

Every olfactory sensory neuron (OSN) is thought to express only a single OR gene from a single allele, which is referred to as singular expression [7,8]. OSNs expressing a given OR are scattered throughout the epithelium but are confined to specific zones [9]. An important feature of the system is that the expression of an OR seems to preclude the expression of additional OR alleles, which is referred to as OR-induced feedback [10,11] and is shown to rely on Adcy3, histone demethylase LSD1 and the unfolded protein response [12–15].

Axons from OSNs that express the same singular expressed OR coalesce into two of the approximately 1800 glomeruli, typically one on the medial and one on the lateral side of the olfactory bulb [16]. The OR itself plays an important role in this impressive developmental task, since mutations that alter single amino acids within an OR sequence or its expression levels reroute axons and shift the position of the glomerulus [5,6,17]. All developmental outcomes by an OR can be substituted by surrogate receptors such as the highly divergent β2-adrenergic receptor [5,18,19]. These experiments have shown that glomeruli do not form an invariant topographical map, which would serve as ‘targets’ for ORs expressed on axons. Instead, glomeruli arise through homotypic interactions, in a context-dependent manner [6]. How ORs and other chemosensory receptors instruct an identity and regulate axonal interactions and glomerular formation has remained unclear. One hypothesis suggests that OR-induced wiring mechanisms fully rely on Gsα and Golfα signalling [18]; ORs would have an indirect role, one of regulating the expression levels of conventional axon guidance and identity molecules. In this model, each OR and OR polymorphism capable of generating unique glomeruli, would have a unique level of basal, agonist-independent activity, which would alter the transcriptional expression of axon guidance molecules such as neuropilin-1. The altered levels of cell surface molecules also regulate anterior–posterior (A-P) targeting of axons: low levels of cAMP result in anterior glomerular positioning and high levels of cAMP result in posterior glomerular positioning [20]. Following A-P positioning, agonist-dependent Golfα signalling in mature OSNs assists in segregating axons into unique glomeruli, by altering the expression levels of cell adhesion molecules, of which Kirrel2, Kirrel3 and BIG2 result in adhesion (attraction of ‘like’ axons), while EphA and ephrinA mediate contact-dependent repulsion (segregation of ‘non-like’ axons) [21–23]. Dorsoventral positioning of glomeruli does not rely on OR signalling, but instead correlates with the anatomical locations of OSNs in the olfactory epithelium and was shown to rely on two sets of repulsive ligands/receptors expressed by OSNs: neuropillin2/Sema3F and Slits/Robo2 [24–26].

We now provide evidence for an extended model of OR-mediated axonal wiring. Importantly, our results indicate that ORs also regulate A-P targeting and axonal identity via cAMP-independent mechanisms. In addition, our findings also redefine concepts within the field of OR gene regulation.

## 2. Results

### 2.1. Conditional deletion of Gsα does not affect the axonal targeting of M71 OSNs

**Figure 1.**
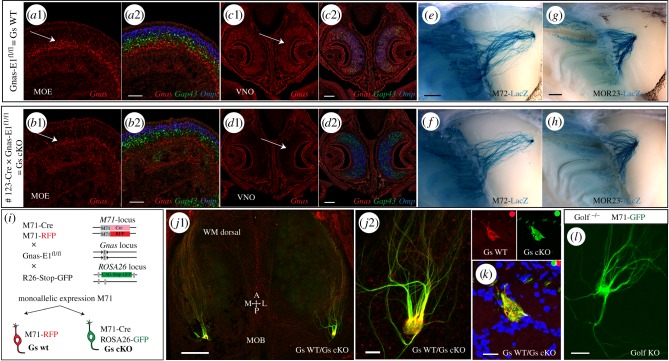
Conditional deletion of Gs in immature OSNs. (*a–d*) Three-colour ISH on coronal sections of PD6 MOE. Riboprobes were used against *Gnas* (red), *Gap43* (green) and *Omp* (blue). (*a*1) MOE *Gnas* is expressed more basal than *Gap43*. (*a*2) Only a fraction of *Gap43* cells colocalize with *Gnas.* (*b*1) Gs cKO mice, *Gnas* expression is no longer observed in *Gap43+* OSNs and remaining expression (*b*2) is more basal. (*c*1) *Gnas* is widely expressed in the VNO of Gnas-E1^fl/fl^ mice ( = Gs WT) and (*c*2) colocalizes with *Gap43* and *Omp*. (*d*1, *d*2) VNO of #123-Cre × Gnas-E1^fl/fl^ mice (i.e. Gs cKO mice), *Gnas* expression is no longer observed. (*e*, *f*) Representative images of X-gal-stained medial wholemounts of M72-LacZ OSNs in (*e*) Gs WT and (*f*) Gs cKO littermates. Bulbs were analysed for PD10 (Gs WT *n* = 10; Gs cKO *n* = 8) and three-week-old (3wo) animals (Gs WT *n* = 10; Gs cKO *n* = 6), no mistargeting was observed. (*g,h*) Representative images of X-gal-stained wholemounts of MOR23-LacZ OSNs in (*g*) Gs WT (*n* = 10) and (*h*) Gs cKO (*n* = 8) littermates (3wo). No mistargeting was observed. (*i*) Mice were crossed to obtain animals carrying all four of the indicated targeted alleles (i.e. quadruple mutant). In the quadruple mutant, M71 OSNs are either: (1) RFP+ and Gs WT or (2) GFP+ and Gs cKO. (*j*1) Wholemount fluorescence of the dorsal bulb in a quadruple mutant described in (*i*) (6wo). RFP+ Gs WT (red) and GFP+ Gs cKO (green) axons converge and comingle (*n* > 10 mice); (*j*2) High magnification view of coalescing axons. (*k*) Coronal sections of the bulb of a quadruple mutant, showing an M71 glomerulus. Gs WT (red) and Gs cKO (green) axons converge and coalesce. DAPI counterstain. (3wo) (*l*) Representative wholemount fluorescence image of an M71-GFP glomerulus in a Golf KO mice (2wo). No mistargeting was observed (*n* = 10 bulbs). MOB, main olfactory bulb; MOE, main olfactory epithelium. Scale bars, 50 μm (*a*2,*b*2,*j*2,*l*), 100 μm (*c*2,*d*2), 500 μm (*j*1,*e*,*g*), 20 μm (*k*).

We aimed to investigate the role of Gsα in the axonal targeting of M71 OSNs. The olfactory epithelium is stratified such that the basal stem cells reside beneath immature neurons that are positive for *growth-associated protein 43* (*Gap43*) and these neurons are below mature neurons that are positive for *olfactory marker protein* (*Omp*). Simultaneous *in situ* hybridization (ISH) using *Gnas* (Gsα), *Gap43* and *Omp* probes revealed detectable expression of *Gnas* in the basal stem cell layer of the developing olfactory epithelium in postnatal day (PD)6 animals that are *Gnas-E1^fl/fl^* (Gs WT, wild-type; figure 1*a* and electronic supplementary material figure S1*d*). Coexpression of *Gnas* was observed in only a fraction of immature neurons expressing *Gap43,* when ORs are first expressed and impart axonal identity to the axons (electronic supplementary material, figure S1*k*). By contrast, *Gnal* (Golfα) expression colocalized with *Omp*, indicating that it was mainly expressed in mature neurons (electronic supplementary material, figure S1*e*). To directly test the role of Gsα signalling in M71 OSNs, we sought to rely on the previously described Gnas-E1^fl/fl^ mice to obtain a conditional knockout allele (cKO) for Gsα [27], since full Gsα KO animals are embryonically lethal. First, we wished to confirm that the Gnas-E1^fl/fl^ mice could be used to delete Gs in OSNs. To this end, we used a transgenic Cre line in which Cre expression is driven by the olfactory epithelium specific #123 promoter. The #123 promoter has been previously shown to be active from an early developmental stage [28] and #123-Cre mice were successfully used to abolish *Sema3F* expression in immature OSNs [24]. We observed that in #123-Cre mice, *Cre* was expressed in all zones and within basal cells, immature and mature neurons (electronic supplementary material, figure S1*a*) and reliably removed the stop fragment in *ROSA26-Stop-tauGFP* reporter mice; mRNA expression of the tauGFP reporter was now observed in basal cells, immature and mature neurons (electronic supplementary material, figure S1*b,c*). All olfactory glomeruli appeared labelled by the *ROSA26-tauGFP* reporter after Cre recombination (electronic supplementary material, figure S1*h*). We next analysed the loss of Gsα from the entire olfactory epithelium in the #123-Cre×Gnas-E1^fl/fl^ (Gs cKO) mice*.* The efficient excision of Gsα was readily observed in the vomeronasal organ (VNO), where the ubiquitous expression seen in WT mice was lost in Gs cKO animals (figure 1*c* versus *d*). In the olfactory epithelium, *Gnas* expression was strongly reduced in *Gap43+* OSNs and was mainly limited to the most basal cells (figure 1*b* and electronic supplementary material, figure S1*f*). Thus, this shows that the Gnas-E1^fl/fl^ mice can be used to eliminate Gsα expression in OSNs. Of note, in the #123-Cre × Gnas-E1^fl/fl^ mice, *Gnal* expression was maintained in mature OSNs with no derepression in immature or basal cells (electronic supplementary material, figure S1*g*). Remarkably, glomeruli appeared normal in the #123-Cre × Gnas-E1^fl/fl^ mice (electronic supplementary material, figure S1*i*) and, by using OR reporters, we observed that M72-LacZ and MOR23-LacZ glomeruli were identical in pattern in both Gs WT and #123-Cre-induced Gs cKO genetic backgrounds (figure 1*e–h*; electronic supplementary material, figure S1*j*). Since deletion of Gs has been previously shown to affect axonal targeting of OSNs [18], this suggests that the #123-Cre promoter is not active early enough during embryonic or neuronal development. Next we set up a mosaic analysis of Gs WT and Gs cKO in OSNs expressing the M71 OR. By using M71-Cre mice and relying on the monoallelic expression pattern of OR genes, we generated a mouse cross containing four different mutant alleles in which M71 OSNs were now either: (i) RFP+ and Gs WT or (ii) GFP+ and Gs cKO (figure 1*i*). No aberrations in axonal targeting were observed in the mutant population of axons (figure 1*j,k*); all axons co-converged and coalesced. Finally, we did not observe any deficits in the glomerular formation of M71-GFP axons in *Gnal−/−* (Golf KO) animals (figure 1*l*; electronic supplementary material, figure S1*l*) and no expression of Gsα protein was observable in Golf KO M71-GFP OSNs (data not shown).

### 2.2. A decrease in G-protein signalling, via the expression of a dominant-negative Gs mutant, does not shift the position of M71 glomeruli

**Figure 2.**
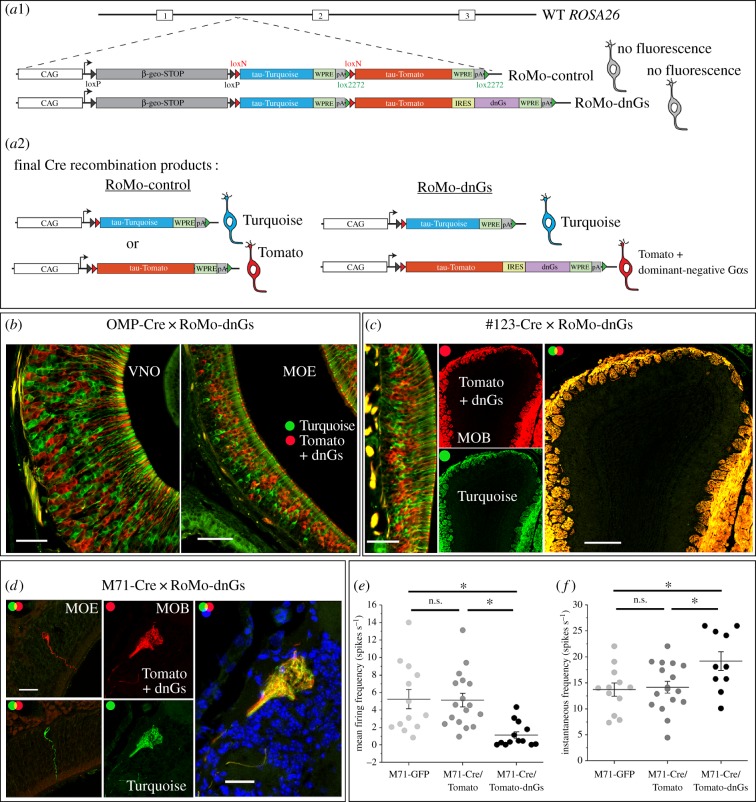
Mosaic expression of a dominant-negative Gs mutant in M71 OSNs impairs basal G-protein signalling but does not affect axonal projections. (*a*1) Schematic overview of *ROSA26* targeted mutations. In RoMo-control and RoMo-dnGs mice, incompatible *lox* sites result in mutually exclusive recombination events leading to a mosaic population. Cre always removes a transcriptional stop cassette flanked by *loxp* sites and randomly recombines either the *loxN* or *lox2272* sites. The final Cre recombination products, which cannot recombine any further, are shown in (*a*2) for RoMo-control and RoMo-dnGs mice. (*b*) Coronal sections through the VNO and MOE of a double heterozygous OMP-Cre × RoMo-dnGs mouse, showing mutually exclusive Turquoise (green) and Tomato (red) fluorescence (3wo). (*c*) Coronal sections through the MOE and MOB of a double heterozygous #123-Cre × RoMo-dnGs mouse, showing mutually exclusive Turquoise (green) and Tomato (red) fluorescence (3wo). (*d*) Coronal sections through the MOE and MOB of a double heterozygous M71-Cre × RoMo-dnGs mouse, showing that M71 axons expressing Tomato and the dnGs mutant (red) converge and comingle with mutually exclusive M71 control axons expressing Turquoise (green) (3wo, *n* = 4), DAPI counterstained. (*e*) Mean and (*f*) instantaneous spontaneous firing frequency recorded through perforated patch-clamp in the current clamp mode from M71 OSNs in M71-GFP mice; M71 OSNs in M71-Cre × RoMo-control mice (M71-Cre/Tomato) and M71 OSNs in M71-Cre × RoMo-dnGs mice (M71-Cre/Tomato-dnGs). While all M71-Cre/Tomato (*n* = 17) and M71-GFP (*n* = 13) OSNs exhibited at least some spikes for each 30 s epoch of recording, 3 out of 13 recorded M71-Cre/Tomato-dnGs OSNs did not exhibit any basal activity (and were also not incorporated in panel *f*). Data are mean ± s.e.m. One-way ANOVA and Tukey post test. (*e,f*). Scale bars, 50 µm (*b,c* left), 250 µm (*c* right), 25 µm (*d*).

To corroborate our Gs cKO and Golf KO results, we envisaged an alternative approach to inhibit basal levels of G-protein signalling by expressing a dominant-negative Gsα (dnGs) mutant that has been reported to efficiently inhibit Gsα signalling [29]. This dnGs mutant is likely to compete with both Gsα and Golfα, thereby reducing basal, agonist-independent G-protein signalling regardless of whether OSNs are expressing Gsα or Golfα. By contrast to the KO experiments, which is a gene ablation in all cells, we wished to set up a competition between cells that contained the dnGs and those that did not, similar to the experiments done with the heterozygous deletion of the X-linked *Cnga2* gene in female mice [30,31]. This type of experiment would cripple one population of cells by dnGs and be directly compared to a WT population of cells in the same animal. Thus, we developed a new approach, the **RO**SA26 **Mo**saic or RoMo system, for mosaic analysis by combining stochastic Cre recombination [32] with gene targeting in the *ROSA26* locus [33] (figure 2*a*). When using cell-type specific promoters to drive Cre expression in RoMo-control mice, we observed a mosaic population of cells, expressing either Turquoise or Tomato (data not shown). This led us to construct a second mouse strain, RoMo-dnGs, where the expression of *Tomato* is now linked to the expression of a dnGs gene via an internal ribosomal entry site (IRES) sequence. Initially, we crossed RoMo-dnGs with OMP-Cre mice to induce recombination in all mature OSNs. Again a mosaic population of either Turquoise+ or Tomato-dnGs+ OSNs was observed in the VNO and main olfactory epithelium (MOE) (figure 2*b*). No apparent segregation of axons was observed in the olfactory glomeruli (data not shown), possibly because the effects of dnGs occurred too late in the development of OSNs. Therefore, to induce recombination in immature OSNs, we crossed RoMo-dnGs mice with our previously described #123-Cre mice. Mosaic expression was observed in the MOE, but both populations of axons converged and comingled into normal glomerular patterns (figure 2*c*). Finally, we sought to investigate how the expression of dnGs affects the identity and basal activity of M71 expressing OSNs by crossing RoMo-dnGs to M71-Cre mice. Mosaic expression was observed with axons that fully converged and comingled (figure 2*d*), indicating that expression of dnGs did not change axonal identity or the glomerular position of M71 OSNs. This suggests that either the inhibition of basal Gs activity did not affect M71 axonal identity or that the dnGs was not functional. It has been reported that spontaneous OSN spiking is fully dependent on the basal signalling of ORs [34]. Therefore, we reasoned that a dnGs-induced knockdown in G-protein signalling would be reflected by a reduction in spontaneous firing. Perforated patch-clamp recordings were performed on dendritic knobs of M71-Cre/Tomato-dnGs+ OSNs using an intact preparation [35]. M71-Cre/Tomato+ OSNs (from M71-Cre × RoMo-control mice) or M71-GFP OSNs were patched as control groups. Interestingly, M71-Cre/Tomato-dnGs+ OSNs exhibited strongly reduced numbers of spontaneous spikes (1.12 ± 0.39 Hz) compared with M71-Cre/Tomato+ (5.13 ± 0.77 Hz) or M71-GFP OSNs (5.25 ± 1.10 Hz; figure 2*e*; one-way ANOVA: *F* = 7.96065; *p* = 0.00123). The instantaneous firing frequency (IF) between these groups also differed (one-way ANOVA: *F* = 4.32191; *p* = 0.02078), with M71-Cre/Tomato-dnGs OSNs having a significantly higher IF (19.2 ± 1.8 Hz) than M71-Cre/Tomato+ (14.2 ± 1.0 Hz) and M71-GFP neurons (13.7 ± 1.3 Hz; figure 2*f*), indicating that M71-Cre/Tomato-dnGs OSNs have a higher tendency for bursting. The basal and IF firing rates of M71-Cre/Tomato+ and M71-GFP OSNs were not different. The reduction in spontaneous firing of M71-Cre/Tomato-dnGs OSNs strongly suggests that the dnGs inhibits basal, agonist-independent G-protein signalling and cAMP production. Since axons of Turquoise+ control OSNs and Tomato+ dnGs-affected OSNs coalesced in the dorsal bulb, this suggests that crippling basal G-protein signalling did not affect M71 axonal identity or glomerular positioning.

### 2.3. Increasing G-protein signalling, via the expression of a constitutively active Gs mutant, can modulate the M71 axonal identity but not the resulting position of its glomeruli

**Figure 3.**
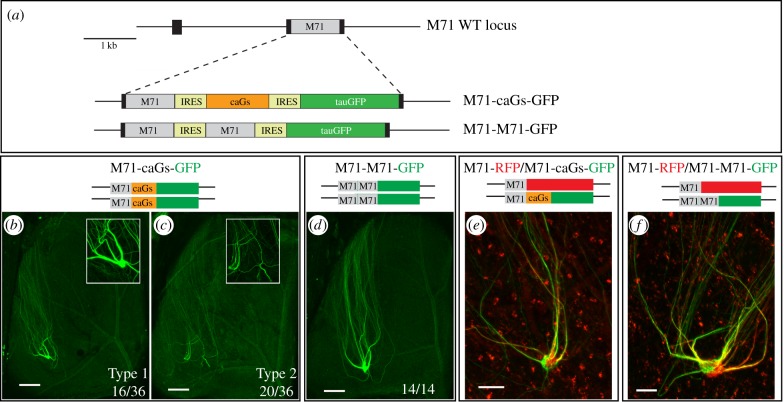
Coexpression of a constitutively active Gs mutant in M71 OSNs does not induce a posterior shift of glomeruli. (*a*) Schematic overview of the targeted *M71* mutations. (*b,c*) Wholemount intrinsic GFP fluorescence of the dorsal bulb (3wo), showing projection sites of lateral M71-caGs-GFP axons (green). (*b*) Glomerular formation is normal (16/36 bulbs). (*c*) Axons project to the correct A-P position, but do not converge into glomeruli (20/36 bulbs). (*d*) Wholemount intrinsic GFP fluorescence in M71-M71-GFP mice (3wo). M71-M71-GFP axons always formed glomeruli (14/14 bulbs). (*e*) Wholemount intrinsic GFP and RFP fluorescence in M71-RFP × M71-caGs-GFP mice (3wo, *n* > 10). M71-RFP (red) and M71-caGs-GFP (green) axons converge, but segregate and form compartmentalized glomeruli. (*f*) Wholemount GFP and RFP fluorescence in M71-RFP × M71-M71-GFP mice (3wo, *n* > 10). M71-RFP (red) and M71-M71-GFP (green) axons converge, but segregate and form compartmentalized glomeruli. Scale bars, 250 µm (*b,c,d*), 50 µm (*e,f*).

We have already addressed the consequences of downregulating G-protein signalling. Now, we wished to assess how axon targeting is affected by an increase in G-protein signalling in M71 neurons. Therefore, we generated the M71-caGs-GFP strain, in which the *M71* locus was engineered to coexpress M71 together with a constitutively active Gs mutant (caGs) and a GFP reporter via a tricistronic mRNA (figure 3*a*). In homozygous M71-caGs-GFP mice, 16 out 36 bulbs showed a lateral GFP+ glomerulus at an A-P position comparable to that of WT M71 (figure 3*b*; Type 1 convergence). Remarkably, in the remainder of bulbs (55%), axons failed to converge into a glomerulus (figure 3*c*), indicating that glomerular formation was disrupted. Importantly, by crossing in the M71-RFP allele, we observed that M71-caGs-GFP axons converged with M71-RFP axons (figure 3*e*). This shows that when M71-caGs glomeruli were formed, they were not posterior to WT M71 glomeruli. This is not because of an intrinsic inability to shift M71 glomeruli posterior, since we have previously reported that the M71::GFP fusion protein induces a clear posterior shift [5]. However, we did observe that M71-caGs-GFP and M71-RFP axons formed compartmentalized glomeruli (figure 3*e*), indicating a subtle change in axonal identity. Together this shows that caGs co-expression did not shift the position of the glomerulus, but did affect axonal identity and glomerular formation.

The frequent failure to form M71-caGs-GFP glomeruli suggests that OR-independent signalling via the caGs mutant could disrupt glomerular formation. However, we cannot exclude that the aberrant wiring resulted from the tricistronic structure of the gene-targeted *M71* locus. By generating the M71-M71-GFP strain, which harbours another tricistronic mutation in the *M71* locus (figure 3*a*), we show that this is unlikely. In M71-M71-GFP mice, where instead of caGs we inserted a second *M71* coding region, the formation of GFP+ glomeruli was always observed (figure 3*d*). Interestingly, M71-M71-GFP and M71-RFP axons also often formed a compartmentalized glomerulus (figure 3*f*), suggesting that the change in M71 protein levels was sufficient to change the identity of the axons.

### 2.4. ORs can regulate A-P targeting via cAMP-independent mechanisms

**Figure 4.**
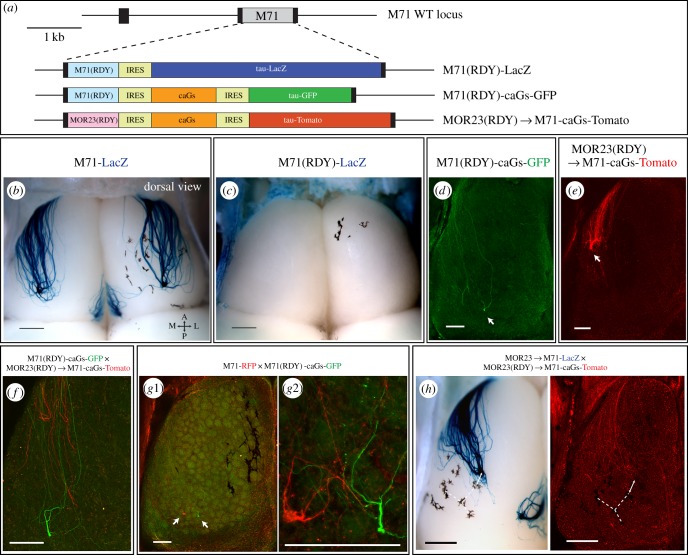
Signalling-deficient M71(RDY) and MOR23(RDY) ORs induce distinct neuronal identities and differentially regulate anterior-posterior targeting of axons. (*a*) Schematic overview of the targeted *M71* mutations. (*b,c*) Dorsal view of X-gal-stained wholemounts of M71-LacZ (3wo) and M71(RDY)-LacZ (PD10) mice. (*d*) Confocal image of a wholemount dorsal bulb from M71(RDY)-caGs-GFP and (*e*) MOR23(RDY) → M71-caGs-Tomato homozygous mice (PD10). Axons are visualized by intrinsic GFP or Tomato fluorescence. Arrows indicate the main projection sites of lateral axons. (*f*) Wholemount fluorescence in M71(RDY)-caGs-GFP × MOR23(RDY) → M71-caGs-Tomato mice (PD10). M71(RDY)-caGs-GFP axons (green) project more posterior and do not fasciculate with MOR23(RDY) → M71-caGs-RFP axons (red). (*g*1, high-magnification view in *g*2) Wholemount fluorescence in M71-RFP × M71(RDY)-caGs-GFP mice (PD10). M71-RFP (red) and M71(RDY)-caGs-GFP (green) axons project to a similar A-P position. (*h*) Wholemounts of MOR23 → M71-LacZ × MOR23(RDY) → M71-caGs-Tomato mice (PD10) were X-gal-stained (left) after confocal imaging (right). MOR23 → M71-LacZ (blue, X-gal) and MOR23(RDY) → M71-caGs-Tomato (red, intrinsic fluorescence) axons project to a similar A-P position (pigmentation is used to create anchor points for reference). Scale bars, 500 µm (*b,c,h*), 250 µm (*d,e,f*,*g*1,*g*2).

Our results indicate that Gs/Golf signalling and cAMP production are not the main drivers of A-P targeting in M71 OSNs. To further corroborate this, we wished to set up an experimental system in which OSNs would express different ORs but have the same level of G-protein signalling. If such axons would not coalesce, this would be a clear indication of an OR-instructed identity, independent of the levels of G-protein signalling. To achieve this, we thought to compare M71 OSNs in which the coding sequence (CDS) was exchanged with distinct signalling-deficient ORs and where G-protein signalling was rescued via expression of the caGs mutant. To induce signalling deficiency in ORs, we chose to mutate the highly conserved DRY motif, which is critical for regulating GPCR conformational states [36]. Mutating the E/DRY residues into REY or RDY has been previously shown to abolish G-protein signalling for Rhodopsin [37], CXCR4 [38] and the rat I7 OR [20]. To confirm that these mutations would indeed cause a loss of function phenotype, we created a gene-targeted strain in which the M71 DRY motif was replaced by RDY (D121R;R122D) along with an IRES-taulacZ reporter, M71(RDY)-LacZ (figure 4*a*). In contrast to M71-LacZ axons (figure 4*b*; electronic supplementary material, figure S2*a*), M71(RDY)-LacZ axons showed poor outgrowth, typically did not reach the cribiform plate and were not observed on the dorsal bulb (figure 4*c*; electronic supplementary material, figure S2*b*,*c*). Furthermore, the number of M71(RDY)-LacZ neurons rapidly decreased over time (electronic supplementary material, figure S2*d*) and by PD21 their numbers were 10-fold lower when compared with M71-LacZ neurons in age-matched animals (electronic supplementary material, figure S2*e*). This dramatic phenotype indicates that G-protein signalling in M71 OSNs is necessary when competing with normal OSNs.

Now we were in a position to test two additional mouse strains: M71(RDY)-caGs-GFP and MOR23(RDY) → M71-caGs-Tomato, in which the RDY mutant OR is coexpressed with a caGs to rescue G-protein signalling (figure 4*a*). Furthermore, in the MOR23(RDY) → M71-caGs-Tomato strain, the *M71* CDS is replaced by a DRY → RDY mutant of the *MOR23* CDS. The M71(RDY)-caGs-GFP and MOR23(RDY) → M71-caGs-Tomato OSNs thus express different ORs—M71(RDY) versus MOR23(RDY)—but should have the same level of G-protein signalling (derived from the OR-independent caGs mutant). Coexpression of caGs rescued axonal projections to the bulb for both M71(RDY)-caGs-GFP and MOR23(RDY) → M71-caGs-Tomato OSNs (figure 4*d,e*). The number of caGs-rescued OSNs remained very low (electronic supplementary material, figure S2*e*) and as a consequence glomerular formation was often inefficient [39]. In mice that were only heterozygous for one of the mutant alleles, axons projected to the expected A-P region, but glomerular formation was often not observed (e.g. Tomato+ axons figure 4*f,h*). These findings are also in line with what was seen for M71-caGs mice, where expression of caGs often disrupted glomerular formation (figure 3*c*). Importantly, however, M71(RDY)-caGs and MOR23(RDY) → M71-caGs axons projected to fundamentally different A-P regions (figure 4*d,e*). The differential identities of M71(RDY)-caGs-GFP and MOR23(RDY) → M71-caGs-Tomato axons were confirmed in a mixed cross, where coalescence was never observed between GFP+ and Tomato+ axons, and where GFP+ axons extended more posterior (figure 4*f*). Remarkably, M71(RDY) axons projected to the same A-P region as WT M71 axons, as was seen in M71-RFP × M71(RDY)-caGs-GFP mice (figure 4*g*). Similarly, MOR23(RDY) → M71-caGs-Tomato axons projected very close to WT MOR23 → M71-LacZ axons, which was anterior to M71 (figure 4*h*). Together this shows that M71(RDY)-caGs and MOR23(RDY)-caGs axons project to distinct A-P regions on the bulb, very close to the targeting site of their respective WT receptors. It is important to note that for both populations the caGs is expressed from the same locus with a similar tricistronic strategy. This suggests that ORs can regulate axonal targeting via cAMP-independent mechanisms.

### 2.5. G-protein signalling is critical for neuronal maturation and competition

**Figure 5.**
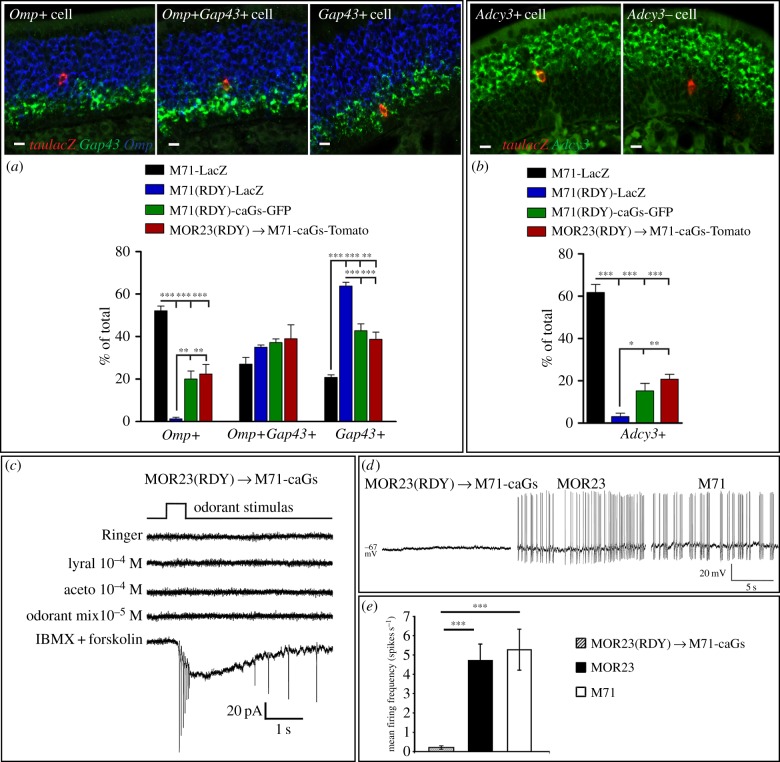
Neuronal maturation and activity are lost in OSNs expressing G-protein coupling mutant ORs. (*a*) Three-colour ISH on coronal sections of the MOE (PD21). Probes were used against *Omp* (blue), *Gap43* (green) and: *taulacZ* (red*, tau* is of bovine origin) for labelling M71-LacZ and M71(RDY)-LacZ OSNs; *tauGFP* for labelling M71(RDY)-caGs-GFP OSNs; *tauTomato* for labelling MOR23(RDY) → M71-caGs-Tomato OSNs (red). Examples are shown of a *OMP+*, *OMP+Gap43+* and *Gap43+* M71 OSN. The percentage of these populations within the total counted cells (% of total) was quantified at PD21 (mean ± s.e.m., *n* = 3). (*b*) Two-colour ISH on coronal sections of the MOE. Riboprobes were used against *Adcy3* (green) and OSN markers (red) as explained in (*a*). Examples are shown for *Adcy3+* and *Adcy3−* M71 OSNs. The percentage of *Adcy3+* and *Adcy3−* OSNs within the total counted cells were quantified at PD21 (mean ± s.e.m., *n* = 3). (*c*) Representative traces of patch-clamp recordings in a MOR23(RDY) → M71-caGs OSN under the voltage-clamp mode. Out of 17 recorded OSNs, 13 responded to IBMX+forskolin. However, these cells did not respond to Ringer solution, lyral, acetophenone (aceto) or a mix of odorants. (*d*) Representative traces of spontaneous activities recorded through perforated patch-clamp in the current clamp mode in MOR23(RDY) → M71-caGs-Tomato or MOR23-GFP or M71-GFP OSNs. Out of 21 recorded MOR23(RDY) → M71-caGs-Tomato OSNs, 14 did not exhibit any spontaneous action potentials. (*e*) Mean spontaneous firing frequency for MOR23(RDY) → M71-caGs (*n* = 21), MOR23 (*n* = 16) and M71 (*n* = 13) OSNs. Data are mean ± s.e.m. One-way ANOVA and Tukey post test. Scale bars, 10 µm (*a,b*). One-way ANOVA and Newman–Keuls post test (*a,b*), *p < 0.05, **p < 0.01, ***p < 0.001.

Are DRY → RDY mutations in ORs blocking G-protein signalling? We observed that M71(RDY)-LacZ cells resided very basal in the epithelium suggesting that these cells were not mature and might not contain necessary signal transduction components to assess G-protein signalling. Thus, we used ISH to determine the percentage of *Omp+* mature, *Omp+Gap43+* intermediate and *Gap43+* immature cells in normal and RDY strains of mice: M71-LacZ, M71(RDY)-LacZ, M71(RDY)-caGs-GFP and MOR23(RDY) → M71-caGs-Tomato OSNs (figure 5*a*). An almost complete absence of *Omp+* mature OSNs was observed in the M71(RDY)-LacZ population (figure 5*a*). M71(RDY)-LacZ OSNs also failed to upregulate *Adcy3* expression, a key component of the signalling machinery in mature OSNs (figure 5*b*). By contrast, *Omp+* and *Adcy3+* mature M71(RDY)-caGs and MOR23(RDY) → M71-caGs OSNs were observed, albeit at a lower percentage as compared with WT M71 (figure 5*a,b*). These results suggest that G-protein signalling may be a checkpoint in OSN maturation. Alternatively, the loss in G-protein signalling may render OSNs uncompetitive and they may be quickly eliminated as they mature, which would explain the rarely observed only *Omp+* expressing M71(RDY)-LacZ OSNs.

We next wished to perform physiology to assess OR functionality. Since M71(RDY)-LacZ OSNs remained immature, they would not be suitable for recordings as their signalling machinery may not be functional. We therefore chose MOR23(RDY) → M71-caGs-Tomato OSNs, on which we performed perforated patch-clamp recordings. MOR23(RDY) → M71-caGs-Tomato neurons were stimulated with saturating concentrations of lyral (an MOR23 ligand), acetophenone (an M71 ligand), or a mixture of odorants that activates 66% of randomly patched OSNs in the MOE [40]. In our analysis, we only considered OSNs that responded to a mixture of IBMX and forskolin, which directly activates adenylate cyclases and indicates whether the neurons are mature. Of the 13 recorded OSNs that responded to IBMX + forskolin (out of 17 total), none responded to any of the other stimulants, showing that there is no chemical or mechanical activity in these neurons (figure 5*c*). In addition, MOR23(RDY) → M71-caGs OSNs exhibited almost no spontaneous activity, which is clearly present in WT MOR23 or M71 OSNs (figure 5*d,e*). Four conclusions are delineated: (i) DRY → RDY mutations uncouple ORs from G-protein signalling; (ii) no other ORs were coexpressed in these cells as MOR23(RDY) → M71-caGs OSNs never responded to the mixture of odorants (gene choice remained intact); (iii) spontaneous OSN spiking is indeed fully dependent on the basal signalling of ORs; and (iv) caGs does not elicit spontaneous spiking, indicating that the rescue of MOR23(RDY) OSNs does not require electrical activity.

### 2.6. One neuron–two receptors: OSNs in O/E2-M71-GFP mice coexpress the M71 OR together with an endogenous receptor

Our results show that signalling-deficient ORs can regulate the identity and A-P targeting of M71 axons, suggesting that this did not rely on differences in OR-induced cAMP levels. However, it may be argued that the M71(RDY) and MOR23(RDY) coding sequences differentially affect mRNA stability, thereby resulting in different caGs protein levels in M71(RDY)-caGs compared to MOR23(RDY)-caGs OSNs. To resolve these issues, we reasoned that forcing the coexpression of a WT OR in all OSNs may also rescue the maturation and axonal projections of M71(RDY) neurons. Furthermore, this might uniformly increase G-protein signalling in all OSNs, allowing a direct comparison of the A-P targeting of M71(RDY) with other ORs. Based on previous experiments using ubiquitously activated ORs via the ttA/tetO system [41–43], it was unclear whether it was *a priori* possible to coexpress a second OR in all OSNs. In theory, OR-mediated negative feedback mechanisms would prevent endogenous gene expression or endogenous genes would silence the expression of the ectopic OR [41,42].

**Figure 6.**
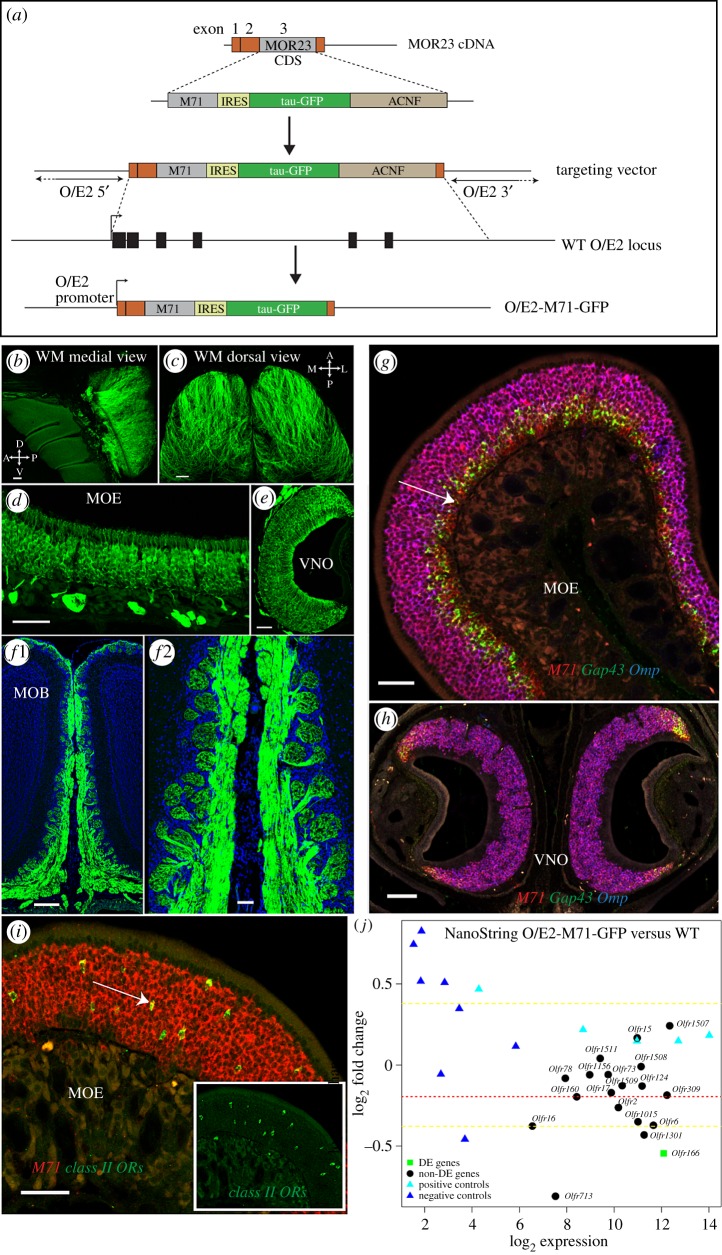
OSNs in O/E2-M71-GFP mice ubiquitously express M71 together with an endogenous OR. (*a*) Schematic of the O/E2-M71-GFP mutation. (*b,c*) Wholemount (WM) intrinsic GFP fluorescence of the medial and dorsal olfactory bulb in O/E2-M71-GFP^+/−^ mice (3wo). (*d–f*) Intrinsic GFP fluorescence in coronal sections of the (*d*) MOE, (*e*) VNO and (*f*1, high magnification in *f*2) MOB of O/E2-M71-GFP^+/−^ mice (3wo). (*g,h*) Three-colour ISH against *M71* (red), *Gap43* (green), *Omp* (blue) in the (*g*) MOE and (*h*) VNO of O/E2-M71-GFP^+/−^ mice (3wo). Arrow shows an example of an OSN expressing M71 before the onset of Gap43. (*i*) Two-colour ISH in the MOE of O/E2-M71-GFP^+/−^ mice, using probes against *M71* (red) and a mix of four different class II OR (see the electronic supplementary material) genes (green and inset). Arrow shows an example of a double positive cell. (*j*) NanoString RNA analysis of whole olfactory mucosa samples collected from six WT and six O/E2-M71-GFP heterozygous littermates (PD25), using a code set against 19 ORs. The values in the *y*-axis represent the log_2_ of the fold change of mutant versus WT (i.e. *M* value). Positive values represent an increase in expression, while negative values are a reduction. The values in the *x*-axis represent the log_2_ of the normalized NanoString counts, which is a measure for both the expression level of the OR and the binding efficiency of the probe. To test for significance, tTreat analysis was used, with a fold change threshold set at 1.3. Differentially expressed genes are indicated as green squares. Red stippled line: the average *M* value of all tested OR genes; Yellow stippled lines: the chosen fold change threshold (set at 1.3, in log_2_ scale = 0.38). Positive controls (cyan triangles) and negative controls (blue triangles) for NanoString operation are distributed correctly as per manufacturer. Scale bars, 250 µm (*b*,*c*,*f*1), 50 µm (*d*,*e*,*f*2,*g*,*i*), 100 µm (*h*).

**Figure 7.**
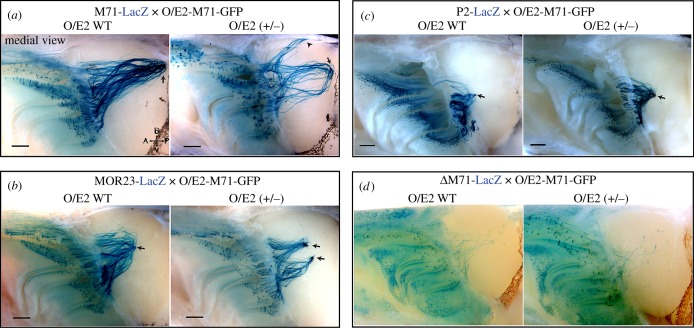
O/E2-M71 is co-expressed with endogenous OR genes. (*a–d*) Wholemount X-gal staining of medial or dorsal bulbs of the indicated crosses. Images on the left are control O/E2 WT littermates, on the right are O/E2-M71-GFP heterozygous animals. M71-LacZ (3wo), MOR23-LacZ (3wo), P2-LacZ (3wo) and ΔM71-LacZ (5wo) mutations are heterozygous; a similar ΔM71-LacZ innervation was also observed in 8wo mice (data not shown). The number of half-bulbs (medial) with two or more glomeruli were quantified for M71: 0/8 in O/E2 WT and 7/14 in O/E2 (+/−); and MOR23: 0/8 in O/E2 WT and 3/6 in O/E2 (+/−). Scale bars, 500 µm.

In an attempt to express an OR in all OSNs, we started from the *MOR23* cDNA (containing exons 1–3, Tg3'Δ, see [44]), where we removed the *MOR23* promoter and *MOR23* coding region and replaced it with an M71-IRES-tauGFP-ACNF cassette (figure 6*a*). This was subsequently cloned into the O/E2 targeting vector (TV) [45] and used to replace the first 6 exons of the *O/E2* gene via gene targeting. In the resulting O/E2-M71-GFP strain, expression of an M71-IRES-tauGFP cDNA is placed under the control of the native *O/E2* promoter, which is prominently expressed in all OSNs [45]. A widespread and bright intrinsic GFP fluorescence was seen in the MOE and VNO of O/E2-M71-GFP mice (figure 6*b–e*). All OSNs appeared GFP+, as did the glomeruli, which were homogeneously labelled and showed a normal morphology (figure 6*f*). Importantly, *M71* mRNA was robustly observed in the whole MOE and VNO of O/E2-M71-GFP mice (figure 6*g,h*; electronic supplementary material, figure S3*a*,*c*). O/E2 expression is typically observed prior to *Gap43,* at a very immature neuronal stage of OSNs. *M71* expression from O/E2-M71-GFP mice was also seen in this early OSN stage (figure 6*g*). The MOE of O/E2-M71-GFP mice had a normal multi-layered structure and thickness, comparable to that of O/E2 WT control animals (electronic supplementary material, figure S3*b,d*). Interestingly, it was evident that expression of *M71* via the *O/E2* promoter did not suppress endogenous OR expression. ISH showed coexpression of *M71* with other unrelated class II OR genes (figure 6*i*). Next, NanoString was used to assess the expression of a random selection of OR genes in the MOE of O/E2-M71-GFP(+/−) and O/E2-WT littermates (figure 6*j*). Of the 19 OR genes tested, only one transcript showed a significant but small downregulation, showing that the majority of ORs had comparable expression levels in the two groups. In addition, the zonal expression of endogenous ORs in the MOE was not altered in O/E2-M71-GFP mice, as illustrated by crossing the mice to the M71-LacZ, MOR23-LacZ and P2-LacZ strains (figure 7*a–c*). As reported previously, OSNs that choose an OR locus that does not contain an OR gene—such as the ΔM71-LacZ allele where the M71 CDS is deleted—coexpress additional OR genes [5]. Therefore, the projection of lacZ+ axons in ΔM71-LacZ mice depends on the guidance properties of the other ORs, resulting in a divergent pattern of projections (figure 7*d*). Interestingly, distributed axons entering multiple glomeruli were also observed in ΔM71-LacZ mice crossed to O/E2-M71-GFP (figure 7*d*).

**Figure 8.**
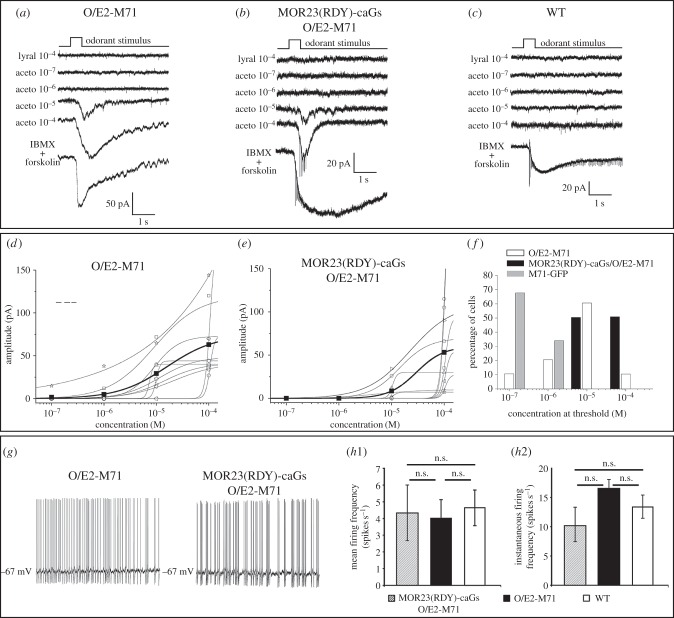
Ectopically expressed O/E2-M71 elicits both agonist-dependent and -independent neuronal activities. (*a–c*) Representative traces of perforated patch-clamp recordings in the voltage-clamp mode in response to the indicated ligands in (*a*) random OSNs or (*b*) MOR23(RDY) → M71-caGs-Tomato OSNs in O/E2-M71-GFP heterozygous mice; (*c*) random OSNs in a WT mouse. (*d,e*) Dose–response curves of acetophenone stimulated (*d*) random OSNs or (*e*) MOR23(RDY) → M71-caGs-Tomato OSNs in O/E2-M71-GFP heterozygous mice. The curve for the average is in black. (*f*) A threshold frequency histogram for random OSNs (white bar, *n* = 10) or MOR23(RDY) → M71-caGs-Tomato OSNs (black bar, *n* = 10) in O/E2-M71-GFP heterozygous mice, and M71-GFP OSNs in O/E2 WT mice (grey bar, *n* = 6). This histogram represents the number of cells with a detection threshold in the 10^−7^, 10^−6^, 10^−5^ or 10^−4^ M range of stimulating concentration. (*g*) Representative traces of spontaneous activities recorded through perforated patch-clamp in the current clamp mode from random OSNs or MOR23(RDY) → M71-caGs-Tomato OSNs in O/E2-M71-GFP heterozygous animals. (*h*1) Mean spontaneous firing frequency and (*h*2) IF for the same OSN types described in (*a–c*). *n* = 6, 13, 10, respectively. Data are mean ± s.e.mb. One-way ANOVA and Tukey post test.

To investigate whether the ectopic M71 in O/E2-M71-GFP mice is functional and contributes to signalling and neuronal activity, we performed patch-clamp recordings on the MOE of O/E2-M71-GFP mice. In addition, we also crossed in the MOR23(RDY) → M71-caGs-Tomato mutation. In the resulting cross, we patched the dendritic knobs of random OSNs that express an unknown endogenous OR (GFP+ Tomato−) or MOR23(RDY) → M71-caGs OSNs (GFP+ Tomato+) and measured their responses to varying concentrations of the M71 ligand acetophenone. All randomly patched OSNs (*n* = 10) and MOR23(RDY) → M71-caGs OSNs (*n* = 10) responded to acetophenone (figure 8*a,b*). While responses were heterogeneous, most O/E2-M71-GFP neurons responded to concentrations above 10^−5^ M acetophenone (figure 8*d,e*). Responses to acetophenone were not widespread in the normal WT MOE, as in O/E2 WT littermates only 1 in 10 patched OSNs responded (figure 8*c*). As a reference we also patched WT M71 OSNs in M71-GFP mice and plotted a threshold frequency histogram, which represents the percentage of cells that responded to a specific threshold concentration (figure 8*f*). This showed that O/E2-M71-GFP neurons were less sensitive to acetophenone than M71-GFP OSNs. Importantly, O/E2-M71-GFP expression also rescued the spontaneous firing of MOR23(RDY) → M71-caGs OSNs, which now exhibited mean and instantaneous firing frequencies in the same range as that of randomly patched OSNs (figure 8*g,h*). Since without O/E2-M71-GFP expression MOR23(RDY) → M71-caGs OSNs exhibit no spontaneous firing (figure 5*e*), this shows that the ectopic M71 contributes to basal, agonist-independent, spiking in OSNs, while caGs does not.

### 2.7. O/E2-M71-GFP rescue experiments confirm that the A-P targeting of ORs and surrogate receptors can be driven by cAMP-independent mechanisms

**Figure 9.**
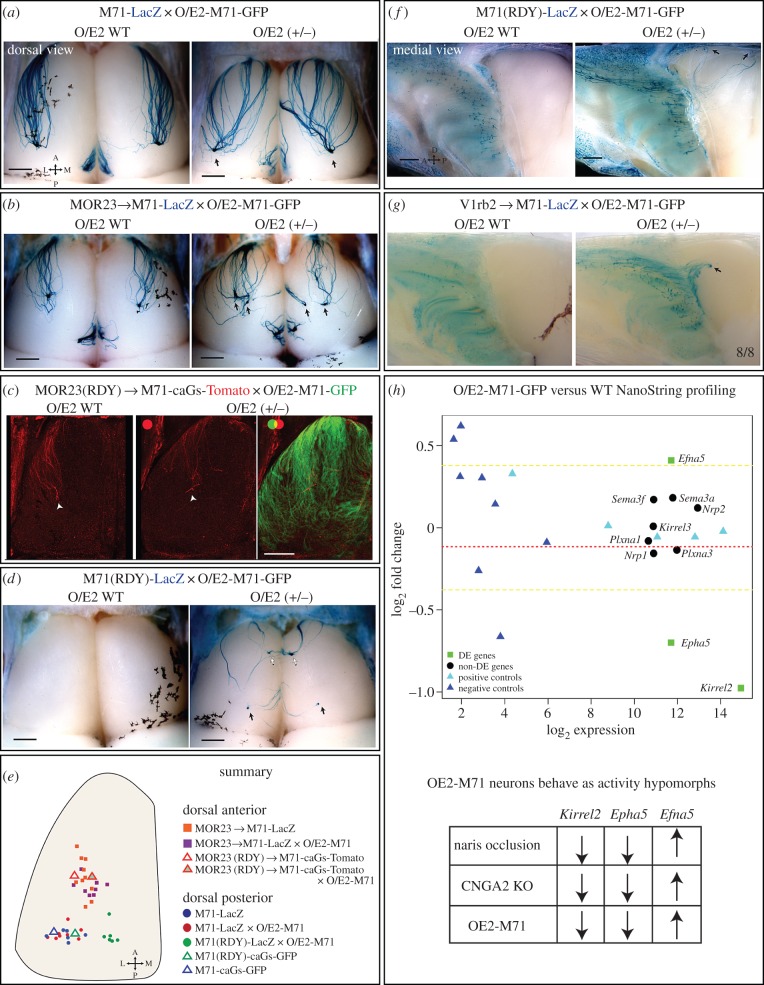
O/E2-M71-GFP rescue experiments show that the anterior–posterior targeting of M71 OSNs is not correlated with the levels of G-protein signalling. (*a,b,d,f,g*) Wholemount X-gal staining of dorsal or medial bulbs of the indicated crosses (PD10–PD21). Images on the left are control O/E2 WT littermates, on the right are O/E2-M71-GFP^+/−^ heterozygous animals. Arrows highlight glomeruli and axonal projections, respectively. White arrows in (*d*) indicate ectopic anteromedial glomeruli. *M71* mutations: (*a,b,g*) heterozygous; (*c,d*) homozygous. (*c*) Wholemount intrinsic GFP and Tomato fluorescence of the dorsal bulb. Arrow indicates the projection site of lateral MOR23(RDY) → M71-caGs axons (red). O/E2-M71-GFP intrinsic fluorescence is shown in green. (*e*) Schematic diagram of the dorsal bulb. Coloured dots represent approximate coordinates of individual lateral glomeruli of the indicated strains. Triangles represent the A-P domain to which the caGs expressing OSNs of the indicated strains project. (*h*) NanoString RNA analysis of whole olfactory mucosa samples collected from six WT and six O/E2-M71-GFP heterozygous littermates (PD25). The *y*-axis represent the log_2_ of the fold change of mutant versus WT (i.e. *M* value), the *x*-axis represents the log_2_ of the normalized NanoString counts. To test for significance tTreat analysis was used, with a fold change threshold set at 1.1. Differentially expressed genes are indicated as green squares. Red stippled line: the average *M* value of all tested OR genes. For reference, the yellow stippled lines are set at a fold change of 1.3 (or 0.38 in log_2_ scale). Positive controls (cyan triangles) and negative controls (blue triangles) for NanoString operation are distributed correctly as per manufacturer. As indicated in the table, the changes in *Kirrel2*, *Epha5* and *Efna5* gene expression in O/E2-M71-GFP mice resemble the published gene expression changes in CNGA2 KO and naris occluded animals. Scale bars, 500 µm.

We wondered how the ectopic M71 expression would affect M71-LacZ or MOR23 → M71-LacZ axonal projections. In O/E2-M71-GFP mutants, M71-LacZ+ and MOR23 → M71-LacZ OSNs formed glomeruli in the correct A-P position (figure 9*a,b*). However, M71 axons took altered routes on the bulb and traversed through regions were they are normally excluded and often formed an additional glomerulus at the same A-P position (figure 9*a,b*; electronic supplementary material, figure S4).

We next tested the consequence of adding M71 expression to signalling-deficient M71(RDY) OSNs. O/E2-M71-GFP expression rescued axon outgrowth of M71(RDY)-LacZ OSNs, which formed two points of coalescence on the dorsal bulb (figure 9*d,f*). An ectopic convergence point was observed in an anteromedial region of the dorsal bulb, where both medial and lateral projecting axons co-converged (figure 9*d*, white arrow). The lateral M71(RDY) glomerulus was observed at the same A-P region as WT M71 glomeruli (figure 9*d*, black arrow), which was clearly more posterior than MOR23 → M71-LacZ or MOR23(RDY) → M71-caGs-Tomato axons (figure 9*b,c,e*). Importantly, in O/E2-M71-GFP mice, M71(RDY)-LacZ OSNs have lower levels of basal G-protein signalling than MOR23 → M71-LacZ OSNs or MOR23(RDY) → M71-caGs-Tomato OSNs. Indeed, while in M71(RDY)-LacZ OSNs only the O/E2-derived ectopic M71 contributes to cAMP production, in the latter two populations this is combined with cAMP stemming from the WT MOR23 receptor or the caGs mutant, respectively. Therefore, despite having lower levels of cAMP, M71(RDY) axons project more posterior than MOR23 → M71-LacZ or MOR23(RDY) → M71-caGs-Tomato OSNs. This again highlights that in M71 or M71-swapped OSNs A-P targeting relies on the expressed OR but not on the levels of cAMP production.

Can we ascertain that the axonal identity and glomerular targeting of O/E2-M71 × M71(RDY) OSNs is imparted by the M71(RDY) OR or could it be derived from the ectopic O/E2-M71 alone? We have previously reported that in V1rb2 → M71-LacZ mice, where the *M71* CDS is replaced by the vomeronasal receptor *V1rb2*, axonal outgrowth and coalescence is very inefficient [5]. Interestingly, O/E2-M71-GFP expression rescued the axonal projections of V1rb2 → M71-LacZ OSNs, which in all analysed bulbs efficiently formed glomerular-like structures (figure 9*g*). This shows that upon O/E2-M71 rescue, V1rb2 can efficiently substitute for an OR and induce a unique axonal convergence. Importantly, V1rb2 → M71-LacZ axons had a different identity and a convergence point that was positioned much more anterior than M71(RDY)-LacZ axons. Since both M71(RDY) and V1rb2 are GPCRs that cannot couple to Gs/Golf, this confirms that their axonal identity and A-P positioning were shaped by OR-dependent, Gsα/Golfα-independent mechanisms.

It has been suggested that the level of basal, agonist-independent G-protein signalling regulates the expression of *neuropilin-1* (*Nrp1*)*, plexin-A1* (*Plxna1*) *and semaphorin-3A* (*Sema3a*) and that these molecules are the main drivers of A-P targeting [18]. To examine whether in O/E2-M71-GFP mice the expression levels of these and other cell adhesion molecules were affected, we performed NanoString analysis on the MOE of O/E2-M71-GFP mutant versus WT littermates (figure 9*h*). There were no changes in the expression levels of any of the neuropilin, semaphorin or plexin genes tested. Surprisingly, we observed a significant downregulation of *Kirrel2* and *Epha5* together with an increase of *ephrin-A5* in O/E2-M71-GFP mice (figure 9*h*). Remarkably, both signalling impaired CNGA2 KO mice and activity reduced, naris occluded animals are known to have this same expression pattern [21]. This suggests that in O/E2-M71-GFP mice there is a general reduction in the electrical activity of OSNs.

## 3. Discussion

Our results provide a set of profound insights into how the OR regulates the developmental pathway of OSNs, starting from regulation of gene choice, to OSN maturation and finally to axonal identity and glomerular formation.

### 3.1. Negative feedback by OR genes

Forcing the widespread expression of an OR using transgenic approaches has not been straightforward, which has contributed to shaping the current models of OR-induced feedback. To achieve ectopic OR expression, several groups have used the tetO-TTA system using immature/mature neuronal TTA drivers [41–43]. These TTA-induced transgenic ORs are able to suppress the expression of endogenous ORs. Remarkably, it has been suggested that the tetO-driven ORs themselves can be silenced by the endogenous ORs, implying that OR coding regions are targets for feedback mediated suppression [41]. Remarkably, all OSNs in O/E2-M71 mice robustly express M71 together with an endogenous OR, while maintaining their specific glomerular formation. This coexpression shows that OR coding regions, including the 5′ and 3′ UTRs, are not targets for feedback suppression, otherwise the endogenous OR would have blocked O/E2-M71 expression. But why did the O/E2-M71 OR, which was already expressed very early in OSN development, not suppress the endogenous OR? One explanation is that negative feedback does not suppress first choice, but only subsequent choices. The projection pattern of ΔM71 OSNs—where first choice does not lead to the expression of an OR—was similar in an O/E2-M71-GFP background. This may indicate that OSNs that have chosen to express the ΔM71 locus still choose a second OR locus for expression, despite the ubiquitous expression of M71 from the O/E2 promoter. An alternative explanation is that second OR choice is blocked by O/E2-M71, but its expression alone is not able to efficiently coalesce ΔM71-LacZ axons into distinct glomeruli. Further characterization of the expressed ORs in ΔM71 OSNs is needed to resolve this.

An explanation for the lack of suppression is that the O/E2-M71 expression levels do not reach a required threshold level to induce feedback. This would imply that robust expression levels that are sufficient to rescue axon outgrowth and neuronal maturation of OSNs are not sufficient to induce negative feedback. It has recently been proposed that stable OR gene expression requires the recruitment of many *cis*- and *trans*-acting enhancers [15]. It is therefore possible that gene choice remains open until one OR locus is able to recruit a sufficient number of enhancers and achieve a high threshold level of expression. Interestingly, recent reports by Hanchate and colleagues [46] have shown that developing OSNs transition from expressing low levels of multiple ORs to expressing high levels of a single OR. These results fit with the interpretation that sub-threshold expression of an OR early in developing OSNs does not block gene choice. Our results also suggest that the expression of an OR is coupled to OSN maturation, as described below.

### 3.2. G-protein signalling and the expression of a functional OR promote OSN maturation

Our results indicate that OSNs expressing ORs with G-protein coupling mutations in the DRY motif remained in an immature state, were unable to send axons to the bulb and were gradually eliminated from the epithelium. Co-expression of a caGs was able to rescue OSN maturation, with the reappearance of *Omp*+ *Adcy3*+ OSNs and axonal projections on the bulb. One explanation for these observations is activity-dependent competition [30], which would result in the rapid elimination of OSNs expressing signalling-deficient ORs. However, no agonist-dependent or -independent neuronal spiking was observed in caGs-rescued OSNs. This suggests that the spatio-temporal dynamics of cAMP production by the caGs did not reach spike threshold levels. Furthermore, the total number of caGs-rescued OSNs in the MOE remained very low, indicating that they were still being eliminated. Together, this makes it unlikely that the caGs-induced reappearance of mature OSNs was linked to activity-dependent competition. One plausible explanation for the observed caGs rescue is a slow or small accumulation of cAMP in immature OSNs that result in a differentiation/maturation signal to the nucleus. Therefore, the expression of a functional OR that is capable of inducing G-protein signalling may directly promote OSN maturation. Interestingly, reports by Lomvardas and co-workers [13] have shown that ORs induce Adcy3 expression via Perk and ATF5 signalling. Adcy3 subsequently downregulates Lsd1 expression, thereby stabilizing OR gene choice [14]. Since the expression of a functional OR, coupled to G-protein signalling, promotes maturation and Adcy3 expression, this could further help in stabilizing OR gene choice. An apparent benefit would be an increased ability to filter out the many dysfunctional OR genes in the repertoire, since signalling-deficient ORs would be less efficient in inducing maturation and Adcy3 upregulation.

The rescue of OSNs by O/E2-M71 is also likely to involve a permissive signal that promotes OSN maturation or survival. Surprisingly, however, OE2-M71 OSNs also showed alterations in the expression of activity-dependent axon guidance molecules that are normally suppressed or enhanced in naris occlusion or CNG2A KO backgrounds [21]. Thus, the rescue of axonal outgrowth in M71(RDY) → M71-LacZ and V1rb2 → M71-LacZ in O/E2-M71-GFP mice may be further enhanced through a reduction in activity-dependent competition, as has been observed in male CNGA2 KO mice (−/0) or naris occluded CNGA2 (+/−) female mice [30]. Why would the ectopic expression of M71 result in a general reduction of activity? We speculate that the ectopic M71 competes for space with the endogenous ORs in the cilia, thereby reducing the sensitivity of OSNs for most odours except M71 ligands. This would concomitantly result in a global reduction in OSN activity.

We show that mosaic Gsα expression using M71-Cre does not result in a differential A-P targeting of WT and Gs cKO axons. This does not strictly mean that Gs is not involved in the development or axonal targeting of M71 OSNs, but rather that the levels of Gsα expression can vary in convergent populations of axons. It also suggests that deleting Gs shortly after the onset of OR expression does not lead to a differential A-P targeting or segregation of WT and cKO axons. It is possible that Gs deletion occurs too late, for example that Gs protein carried over from the basal stem state to immature OSNs is sufficient to support signalling. In this regard, there are indications that Gs mRNA may have a long half-life [47]. Also, if Cre recombination at the *Gnas* locus is not very efficient, then Gs may be deleted with some delay following Cre onset. In the absence of Gs, low levels of Golf protein may rescue G-protein signalling. However, Golf by itself was not critical, since Golf KO animals retained normal formation of M71 glomeruli in the dorsal bulb consistent with previous reports for P2 [48] and rI7 [18] glomeruli. While keeping some of the aforementioned caveats in mind, based on our mosaic Cre-deletion experiments, we speculate that leaky or low levels of Gs or Golf protein are sufficient to support normal axonal targeting. We favour the interpretation that Gs carried over from basal cells to immature OSNs plays a role in kick-starting signal transduction shortly after OSNs start to express an OR. This would subsequently promote maturation and further upregulation of the signal transduction machinery. In line with this Nakashima and colleagues [18] have shown that simultaneous deletion of both Gs and Golf results in severe targeting defects.

### 3.3. A case for cAMP-independent mechanisms in regulating A-P targeting and axonal identity of M71 or M71-swapped OSNs

The molecular mechanism of how ORs and other chemosensory receptors provide ‘self’ identity, thereby regulating axonal interactions and glomerular formation in the olfactory system, remains unclear. The state of OSN activity has often been ascribed as having a role in axonal wiring [49]. One model for axonal identity is based on OR-specific G-protein signalling that would in turn provide discrete levels of cAMP within each type of OSN [23]. The source of cAMP production would be derived by Gsα signalling within immature OSNs and Golfα signalling within mature OSNs. Interestingly, we now have obtained results which show that for M71 or M71-swapped OSNs, OR-induced A-P targeting also relies on cAMP-independent mechanisms. This conclusion is based on various observations.

If A-P targeting would solely rely on differences in basal cAMP levels, then OSNs with the same level of basal G-protein signalling would project to the same A-P position, irrespective of the expressed OR. Since every OR is suggested to induce different basal activities, we devised a genetic swap experiment, where we expressed different signalling-deficient ORs from the same endogenous locus, and restored activity via receptor-independent G-protein signalling. Importantly, this showed that signalling-deficient M71(RDY) and MOR23(RDY) ORs induced distinct identities and projected axons to different A-P regions on the bulb, close to the cognate WT receptor. These data show that even in the absence of effective coupling of ORs to G proteins, M71(RDY) and MOR23(RDY) ORs were still regulating A-P targeting. Furthermore, M71(RDY) and MOR23(RDY) → M71 axons did not coalesce, showing that they also had different identities. This suggests that both A-P targeting and axonal identity can be regulated via cAMP-independent mechanisms. However, we cannot rule out small differences in mRNA stability, leading to differential caGs protein levels, or that the RDY mutants still possess some difficult to detect residual activity. In this regard, the expression of caGs may provide a permissive environment to allow OSN maturation and axon outgrowth. Under this condition, the coupling of the mutant ORs with the G proteins, albeit ineffective, may still affect glomerular positioning. However, it is questionable if the system would be sensitive enough to detect such small variations in signalling. Moreover, a striking observation, which cannot easily be explained by the aforementioned arguments, was that M71(RDY) and MOR23(RDY) ORs sent axons to the same A-P position as their respective WT receptors.

To further investigate how subtle changes in G-protein signalling would affect axonal identity and A-P targeting in M71 OSNs, we also modified cAMP production through the coexpression of a constitutively active or dominant-negative Gsα mutant. Using a mosaic readout, neither was found to induce a shift in the position of M71 glomeruli. However, one cautionary note is that we do not yet know to what extent the dnGs or caGs mutants modulate basal cAMP production in OSNs. In the case of the dnGs mutant, we used spontaneous electrical activity as a proxy for basal cAMP signalling, which is based on previous observations [34,50]. However, this does not allow us to ascertain the exact level of cAMP inhibition, which will require new experimental approaches. Our results with the Gsα mutant Q227L are in contrast to the changes observed for the rI7 glomeruli, where the same Gs mutant could shift the glomerular position [20]. One explanation may be found in the heterogeneity of OSNs. The existence of different OSN cell types that target to distinct domains on the bulb has been demonstrated [51]. Our work is centred on OSNs that are able to choose the endogenous *M71* locus, while Imai and colleagues [20] use the MOR23 minigene strategy, a transgene expressed in OSNs that are able to choose the endogenous *MOR23* locus. Our results may suggest that the importance of G-protein signalling and cAMP production in axonal wiring may vary depending on the OSN cell type. Alternatively, the difference may simply be due to the transgenic versus gene-targeted approach. The transgenic approach is sensitive to variations in transcription levels, cell-type expression and RNA stability, which can also affect the expression levels of caGs.

Additional evidence showing that ORs can regulate A-P targeting via cAMP-independent mechanisms was provided by the O/E2-M71 rescue experiments. These showed that upon O/E2-M71 rescue, the A-P position of the lateral M71(RDY) glomeruli closely matched that of WT M71, which could not be explained by invoking the levels of G-protein signalling and cAMP production. We also show that upon O/E2-M71 rescue, the V1rb2 receptor can robustly provide for axonal identity and the coalescence of axons. Importantly, the M71(RDY) and V1rb2 glomeruli were in completely different regions of the bulb. This shows that in a permissive environment (via O/E2-M71 expression), the M71(RDY) and V1rb2 genes regulate A-P targeting and axonal coalescence. Since both receptors have a deficiency in coupling to Gs/Golf, this suggests the involvement of cAMP-independent mechanisms.

An important recent discovery was the existence of a developmental critical period in olfactory map formation [52,53]. Work by the Barnea laboratory has shown that tetO-TTA-mediated ectopic expression of MOR28 in a subset of OSNs results in the appearance of multiple transgenic MOR28 glomeruli. Interestingly, if transgene expression is active during early development, endogenous MOR28 axons are found to reroute to additional nearby glomeruli that are co-innervated by transgenic MOR28 axons. This shows that the axonal targeting of OSNs is affected by other OSNs that express the same OR, indicating non-cell autonomous effects [52]. Interestingly, a striking feature of the axonal projections in O/E2-M71 mice was that OSNs expressing a given OR took altered routes on the bulb and often formed multiple glomeruli (see the electronic supplementary material, figure S4 for additional examples). One hypothesis is that due to the global co-expression of M71 in all OSNs, there are now M71-dependent homotypic interactions between axons that express different endogenous ORs. These interactions may allow axons to trail along novel axonal tracts and thus re-route to nearby regions. If this hypothesis were to be correct, it is likely that the M71-induced homotypic interactions are not cAMP-dependent, since the ectopic M71 expression is global and should affect G-protein signalling to the same extent in all OSNs.

### 3.4. A case for cAMP/activity-dependent mechanisms in regulating axonal identity and glomerular formation

Importantly, in an O/E2-M71 background, M71(RDY) axonal projections did not completely mirror those of WT M71. Indeed, the medial M71(RDY) glomeruli were in a new ectopic anterior position. Two independent groups have shown that in Adcy3(−/−) animals, M71 and M72 axons form, besides the expected glomeruli, an additional glomerulus in an anteromedial region [54,55]. Strikingly, the position of the anteromedial Adcy3(−/−) M71 glomeruli closely resembles that of the medial O/E2-rescued M71(RDY) glomeruli. This invites the hypothesis that in the absence of normal G-protein-dependent activity, additional glomeruli can be formed. This activity-dependent exclusion may rely on specific adhesive or repulsive axon guidance molecules such as neuropilin, kirrel and ephrins, the expression of which may be altered or lost in activity mutants [54]. In this regard, the altered axonal projections and mistargeting observed in O/E2-M71 mice, may in part be due to our observed changes in the expression of *Kirrel2*, *Epha5* and *ephrin-A5*.

Furthermore, we observed that M71 and M71(RDY)-caGs axons had distinct identities. It is known that even single amino acid substitutions can alter axonal identity [6]. Importantly, M71 and M71(RDY)-caGs have completely different activity profiles, with M71(RDY)-caGs OSNs resembling activity knockouts. This suggests that the differential axonal identities of these OSNs are shaped by their neuronal activity patterns. The importance of neuronal activity for axonal identity and glomerular segregation has been previously reported. For example, blocking neuronal activity via the forced expression of Kir2.1 leads to the erroneous innervation of multiple glomeruli by neurons expressing the same OR [53]. The segregation of M71 and M71-caGs axons further fits this interpretation. CaGs-induced changes in G-protein signalling may affect the expression of adhesive/repulsive molecules and thereby change axonal identity [23]. Furthermore, while in the presence of a WT allele M71-caGs axons were consistently found to converge with M71 glomeruli, in homozygous mutants glomerular formation of M71-caGs OSNs was frequently disrupted. While the mechanistic reasons are unclear, we speculate that this is due to a reduction of neuronal survival leading to fewer projections present needed to form stable glomeruli [39]. Remarkably, a previous study has shown that the expression of caGs via retroviral vectors can also induce the convergence of axons expressing different ORs [56].

## 4. Conclusion

In conclusion, our results suggest that, besides activity-dependent mechanisms, the A-P targeting and axonal identity of OSNs is also regulated by mechanisms that do not rely on cAMP production. That ORs can regulate axonal wiring via cAMP-independent mechanisms is yet another surprising feature of these highly pluralistic proteins. How do ORs, which are present in olfactory axons [5,57], support axonal wiring without relying on canonical G-protein signalling? Based on our contextual model of axonal coalescence [6], we previously proposed that a possible mechanism of axonal identity is through ORs providing structural identity (a key) to as yet unidentified cofactors, which would mediate homotypic interactions. Recent work also suggests that ORs can instruct adhesive properties in cells [58]. Alternatively, other G-proteins that do not couple via the DRY motif or non-canonical signalling pathways (e.g. Perk) may be involved. These will be interesting starting points for future investigations.

## 5. Material and methods

### 5.1. Gene targeting and experimental animals

Targeting constructs for M71(RDY)-lacZ, M71(RDY)-caGs-GFP, MOR23(RDY) → M71-caGs-Tomato and M71-M71-GFP were generated by modifying the described M71 TV [5]. The O/E2-M71-GFP targeting construct was generated by modifying the described O/E2 TV [45]. The RoMo-control and RoMo-dnGs targeting vectors were generated by modifying the described Gateway-compatible *ROSA26* locus targeting vectors [59].

#### 5.1.1. M71(RDY)-lacZ, M71(RDY)-caGs-GFP, M71-caGs-GFP, MOR23(RDY) → M71-caGs-Tomato

Starting from the 9.2 kb M71 genomic sequence [5], we first generated an M71(RDY)-ACNF and an MOR23(RDY) → M71-ACNF TV. For M71(RDY)-ACNF, this included four point mutations in the *M71* coding region (GACCGC → CGCGAC), resulting in the D121R;R122D substitutions. For the MOR23(RDY) → M71 swap, the 930 bp *M71* coding region was replaced with the 930 bp *MOR23* coding region, which again harboured four point mutations (GATCGT → CGTGAT) resulting in the same D121R;R122D substitutions. An FseI and an AscI restriction site was inserted three nucleotides after the stop codon of the M71(RDY) or MOR23(RDY) CDS, and was followed by an ACNF cassette, which is a *neo* selection cassette, that is self-excised during transmission though the male germ line [60]. The IRES-taulacZ, IRES-caGs-IRES-tauGFP or IRES-caGs-IRES-tautdTomato cassettes were subsequently inserted via directional cloning using the FseI and AscI sites, generating the M71(RDY)-taulacZ-ACNF, M71(RDY)-caGs-tauGFP-ACNF and the MOR23(RDY) → M71-caGs-tautdTomato-ACNF targeting vectors. The constitutively active Gαs mutant (Q227L) is previously described [61]. The M71-caGs-GFP strain was derived by screening targeted M71(RDY)-caGs-GFP embryonic stem (ES) cell clones via restriction fragment length polymorphism and sequencing to find clones in which the 5' homologous recombination event was after the DRY → RDY mutation. For M71-M71-GFP, an IRES-M71-IRES-tauGFP-LNL cassette was inserted via PacI, three nucleotides after the stop codon of *M71* CDS. The LNL *neo* cassette was removed by crossing to EIIA-Cre mice [62].

#### 5.1.2. O/E2-M71-GFP

The O/E2-M71-GFP targeting construct was generated by ligating an AscI M71-IRES-tauGFP-ACNF fragment into the O/E2 TV [45]. This AscI fragment was constructed using the Tg3'Δ construct without the 405 bp MOR23 promoter and with the 930 bp MOR23 CDS replaced with the 930 bp M71 CDS followed by a PacI site. In addition, an IRES-tauGFP-ACNF PacI cassette was cloned into the AscI cassette.

#### 5.1.3. Romo-control and RoMo-dnGs

To generate the RoMo-control and RoMo-dnGs TVs, we modified the previously described attL4-pCAGG-loxP-Bgeo-3xpA-loxP-attR1 Gateway-compatible entry clone (pCAGG entry clone) [59], which was obtained via the BCCM/LMBP Plasmid Collection. A loxN-taumTurquoise-WPRE-pA-lox2272-loxN-tautdTomato-WPRE-pA-lox2272-attL3 (for RoMo-control) or loxN-taumTurquoise-WPRE-pA-lox2272-loxN-tautdTomato-IRES-dnGs-WPRE-pA-lox2272-attL3 (for RoMo-dnGs) cassette was inserted into the pCAGG entry clone via kpnI and NotI directional cloning, which also removed the attR1 site. This entry clone was subsequently inserted into the p*ROSA26-DV3* destination vector [59] provided by BCCM/LMBP using Gateway cloning (LR reaction between attL3-attR3 and attL4-attR4). LR reactions were performed using the Clonase Enzyme Mix (Invitrogen) following the supplier's instructions. β-geo-STOP is a β-*galactosidase–neomycin* fusion gene, followed by three pA sequences, that allows for selection in ES cells and is expressed in the absence of Cre recombination. The dominant-negative Gαs mutant (α3β5/G226A/A366S) is previously described [29] and was kindly provided by Dr Catherine Berlot (Weis Center for Research, USA). mTurquoise [63] was kindly provided by Dr Theodorus W. J. Gadella (University of Amsterdam, The Netherlands). The woodchuck hepatitis virus posttranscriptional regulatory element (*WPRE*) was used to increase expression levels [64].

Gene targeting was performed in E14 ES cells as described [17]. ES cells were injected into C57BL/6 blastocysts. Mice are in a mixed 129/B6 background. All generated mice will be made publicly available via the Jackson Laboratory: M71(RDY)-lacZ: B6;129P2-Olfr151<tm38Mom>/MomJ, stock# 23669; M71(RDY)-caGs-GFP: B6;129P2-Olfr151<tm39(Gnas*)Mom>/MomJ, stock# 23672; MOR23(RDY) → M71-caGs-Tomato: B6;129P2-Olfr151<tm37 (Olfr16*,-Gnas*,-tdTomato)Mom>/MomJ, stock# 22789; M71-caGs-GFP: B6;129P2-Olfr151<tm40(Gnas*)Mom>/MomJ, stock# 24642; M71-M71-GFP: B6;129P2-Olfr151<tm39(Olfr151)Mom>/MomJ, stock# 8092; O/E2-M71-GFP: B6;129P2-Ebf3<tm1(Olfr151)Mom>/MomJ, stock# 8094; RoMo-control: B6;129P2-Gt(ROSA)26Sor<tm3Mom>/MomJ; stock# 18670; RoMo-dnGs: B6;129P2-Gt(ROSA)26Sor<tm2Mom>/MomJ, stock# 18669.

Previously described strains that were used in this study (see also the electronic supplementary material, figure S5) are: M71-LacZ, M71-GFP, MOR23 → M71-LacZ, V1rb2 → M71-LacZ, M72-LacZ, ΔM71-LacZ [5], M71-RFP, M71-Cre, OMP-Cre [65], P2-LacZ [17], MOR23-LacZ and MOR23-GFP [44]. #123-Cre mice [24], Gnas-E1^fl/fl^ mice [27], R26-STOP-tauGFP (ROSA26-CAGS-tauGFP) mice [66] and Golf KO mice [48] were kindly provided by Dr Yoshihiro Yoshihara, Dr Lee Weinstein, Dr Ulrich Boehm and Dr Leonardo Belluscio, respectively.

### 5.2. Wholemount staining and imaging

Wholemount X-gal staining was performed as previously described [17]. The A-P and D-V coordinates of X-gal-stained glomeruli in wholemounts where determined using ImageJ software. Intrinsic fluorescence in wholemounts was acquired with an upright Zeiss LSM710 microscope. Images were collected as *z*-stacks followed by maximum intensity projection to a single image.

### 5.3. ISH and IHC

Mice were anaesthetized and perfused with 4% PFA in PBS. Heads were post-fixed, decalcified, cryoprotected, frozen and sectioned at 12 µm, as described [40]. IHC was performed as described [40]. The following antibodies were used: chicken-anti-βGal (Abcam, ab9361), chicken-anti-GFP (Abcam, ab13970), rabbit-anti-Dsred (Clontech, 632496), donkey-anti-rabbit AF555 (Invitrogen, A31572), goat-anti-chicken AF488 (Invitrogen, A11039). Multi-colour ISH was performed as previously described [67]. For riboprobes that were used, see the electronic supplementary material, table S1. For quantifying the percentage of OSNs at distinct maturation states in figure 5*a,b*, every 10th section was collected from anterior to posterior and 42 sections were analysed per mouse.

### 5.4. Patch-clamp recordings

Animals were allowed access to food and water ad libitum and were kept on a 12 L : 12 D cycle, with a constant temperature.

Patch-clamp recordings were performed as described earlier [35,68]. Briefly, male or female gene-targeted three to five-weeks-old mice were anaesthetized by injection of ketamine HCl and xylazine (150 mg kg^−1^ and 10 mg kg^−1^ body weight, respectively), and then decapitated. The head was immersed in ice cold Ringer's solution, which contained (in millimolar): NaCl 124, KCl 3, MgSO_4_ 1.3, CaCl_2_ 2, NaHCO_3_ 26, NaH_2_PO_4_ 1.25, glucose 15; pH 7.6 and 305 mOsm. The pH was kept at 7.4 by bubbling with 95% O_2_ and 5% CO_2_. The nose was dissected out *en bloc*. The olfactory epithelium attached to the nasal septum and the dorsal recess was removed and kept in oxygenated Ringer. Right before starting the recording session, the entire epithelium was peeled away from the underlying bone and transferred to a recording chamber with the mucus layer facing up. Oxygenated Ringer was continuously perfused at room temperature.

OSNs' dendritic knobs were visualized through an upright microscope equipped with an Olympus DP72 camera and a 40× water-immersion objective. An extra 2-to-4× magnification was achieved by a magnifying lens in the light path. The GFP+ or tdTomato+ labelled cells were visualized under fluorescent illumination. Superimposition of the fluorescent and bright field images allowed identification of the fluorescent cells under bright field, which directed the recording pipettes. Electrophysiological recordings were controlled by an EPC-10 USB amplifier combined with Patchmaster software (HEKA Electronic, Germany). Perforated patch-clamp was performed on the dendritic knobs by including 260 µM nystatin in the recording pipette, which was filled with the following solution (in mM): KCl 70, KOH 53, methanesulfonic acid 30, EGTA 5, HEPES 10, sucrose 70; pH 7.2 (KOH) and 310 mOsm. The junction potential was approximately 9 mV and was corrected in all experiments off-line. For odorant-induced transduction currents, signals were sampled at 20 kHz. Under voltage-clamp mode, the signals were initially filtered at 10 kHz and then at 2.9 kHz.

A seven-barrel pipette was used to deliver stimuli by pressure ejection through a picospritzer (Pressure System IIe, Toohey Company, Fairfield, NJ, USA). The stimulus electrode was placed approximately 20 µm downstream from the recording site. Distance (approx. 20 µm) and pressure (20 psi) were adjusted in order to minimize mechanical responses [69]. Single odorant stimuli were prepared in 0.5 M solution in dimethyl sulfoxide (DMSO) and kept at –20°C. Final solutions were prepared before each experiment by adding Ringer. The odorant mixture consists of 19 compounds in equal molar concentration [40,70]: heptanol, octanol, hexanal, heptanal, octanal, heptanoic acid, octanoic acid, cineole, amyl acetate, (+) limonene, (−) limonene, (+) carvone, (−) carvone, 2-heptanone, anisaldehyde, benzaldehyde, acetophenone, 3-heptanone and ethyl vanilline. Odorant mixture was prepared as a 0.1 M solution in DMSO and kept at −20°C; final solutions at 10^−5^ M for each odorant were prepared before each experiment by adding Ringer. Forskolin, an activator of adenylyl cyclase, was prepared as a 10 mM stock solution in DMSO. IBMX, an inhibitor of phosphodiesterases, was prepared as a 100 mM stock solution in DMSO. Final solution containing 200 µM of IBMX and 20 µM of forskolin was prepared before each experiment by adding Ringer.

Unless specified, all chemicals were from Sigma-Aldrich (St-Quentin Fallavier, France). Lyral was provided as a generous gift from International Fragrances and Flavors (Dijon, France).

Data were analysed using Fitmaster (HEKA). Maximum amplitude of the response and kinetics characteristics was measured. Dose–response curves were drafted and fitted using Origin software (OriginLabs). Statistical analysis (ANOVA, non-paramteric Kolmogorov–Smirnov tests followed by Mann–Whitney *U*-tests for two independent samples) were performed using Origin software (OriginLabs).

### 5.5. NanoString analysis

Total RNA extraction from whole olfactory mucosa was performed as described [71]. One microgram of RNA was used for each assay. Processing of raw counts and determination of differential expression was performed as described [72]. The reference genes used were *Omp* (NM_011010.2)*, Gnal* (NM_177137.4)*, Adcy3* (NM_001159537.1)*, Ano2* (NM_153589.2) and *Cnga2* (NM_007724.2). For the list of examined ORs and axon guidance molecules, see the electronic supplementary material, table S2. The raw NanoString counts for the various genes are included in the electronic supplementary material.

## Supplementary Material

Supplemental files Movahedi et al Open Biology

## Supplementary Material

Nanostring data Movahedi et al Open Biology
