# Supplementary material for: Odorant receptors can mediate axonal identity and gene choice via cAMP-independent mechanisms
Source: Open Biol. 2016 Jul 27;6(7):160018. doi: 10.1098/rsob.160018 (PMC4967819; doi:10.1098/rsob.160018)
Supplement: Supplemental files Movahedi et al Open Biology [file rsob160018supp1.pdf]

Supplemental information inventory:

<u>Supplemental Figures and Legends:</u>

S1 (related to Figure 1): The use of #123-Cre mice for the deletion of Gsα in immature OSNs.

S2 (related to Figures 4 and 5): OSNs expressing G-protein coupling M71 mutant ORs are gradually eliminated from the epithelium and their axons are rarely observed on the bulb.

S3 (related to Figures 6 and 8): O/E2-M71-GFP mice ubiquitously express *M71*.

S4 (related to Figures 7 and 9): O/E2-M71 expression affects axonal projections of OSNs expressing endogenous ORs.

S5 (related to all Figures): Previously described mouse strains with gene-targeted OR mutations and their abbreviations that were used throughout the study.

<u>Supplemental Tables:</u>

S1 (related to Figures 1, 5, 6, S1, and S3): List of ISH probes that were used.

S2 (related to Figures 6 and 9): List of nanostring probes that were used.

Supplemental Figures and tables.

Figure S1. The use of #123-Cre mice for the deletion of Gsα in immature OSNs.

(A) Three-colour ISH on coronal sections of PD6 MOE of #123-Cre x ROSA26(R26)-STOP-tauGFP mice. Riboprobes were used against *Cre* (red-A1,-A2), *Gap43* (green-A2) and *Omp* (blue-A2). *Cre* is expressed before the onset of Gap43. (B, C) Three-colour ISH on coronal sections of PD6 MOE of #123-Cre x R26-STOP-tauGFP mice with probes against bovine *tau* (red-B1, -B2, -C1, -C2), *Gap43* (green-B2, C2) and *Omp* (blue-B2,-C2). Expression of tau is only observed after removal of the loxp flanked transcriptional stop cassette of the R26-STOP-tauGFP allele, which is therefore an indicator of successful Cre recombination. Tau is expressed before the onset of Gap43. (D, F) Three-colour ISH on coronal sections of PD6 Gs WT (D) and Gs cKO (F) MOE with probes against *Gnas* (red-D1,-D2, -F1, -F2), *Gap43* (green-D2, -F2) and *Omp* (blue-D2, -F2). In Gs cKO mice, *Gnas* expression is clearly more basal throughout the whole epithelium, indicating that it is mostly expressed in the basal progenitors. (E, G) Three-colour ISH on coronal sections of PD6 Gs WT and Gs cKO MOE with probes against *Gnal* (red), *Gap43* (green) and *Omp* (blue). (H, I) Intrinsic GFP fluorescence observed in coronal sections of the MOB from #123-Cre x R26-STOP-tauGFP (= Gs WT) in H1 and high magnification H2 and #123-Cre x R26-STOP-tauGFP x Gnas-E1$^{fl/fl}$ (= Gs cKO) mice in I1 and high magnification I2. Intrinsic GFP reporter expression is only seen after Cre recombination. DAPI counterstain. (J) The A-P position of individual M72 (PD10, Gs WT n=8, Gs cKO n=8) and MOR23 (PD21 Gs WT n=8, Gs cKO n=8) glomeruli in Gs WT and cKO animals. (K) Higher magnification of Gs WT, Figure 1A2 (see also D2 above), *Gnas* (red), *Gap43* (green), showing that some *Gap43+* cells express *Gnas* (highlighted with arrows). (L) Lower magnification of posteriorly projecting lateral M71 glomerulus in Golf KO mice (PD11, scale bar: 250μm). The M71-GFP mutation is homozygous.

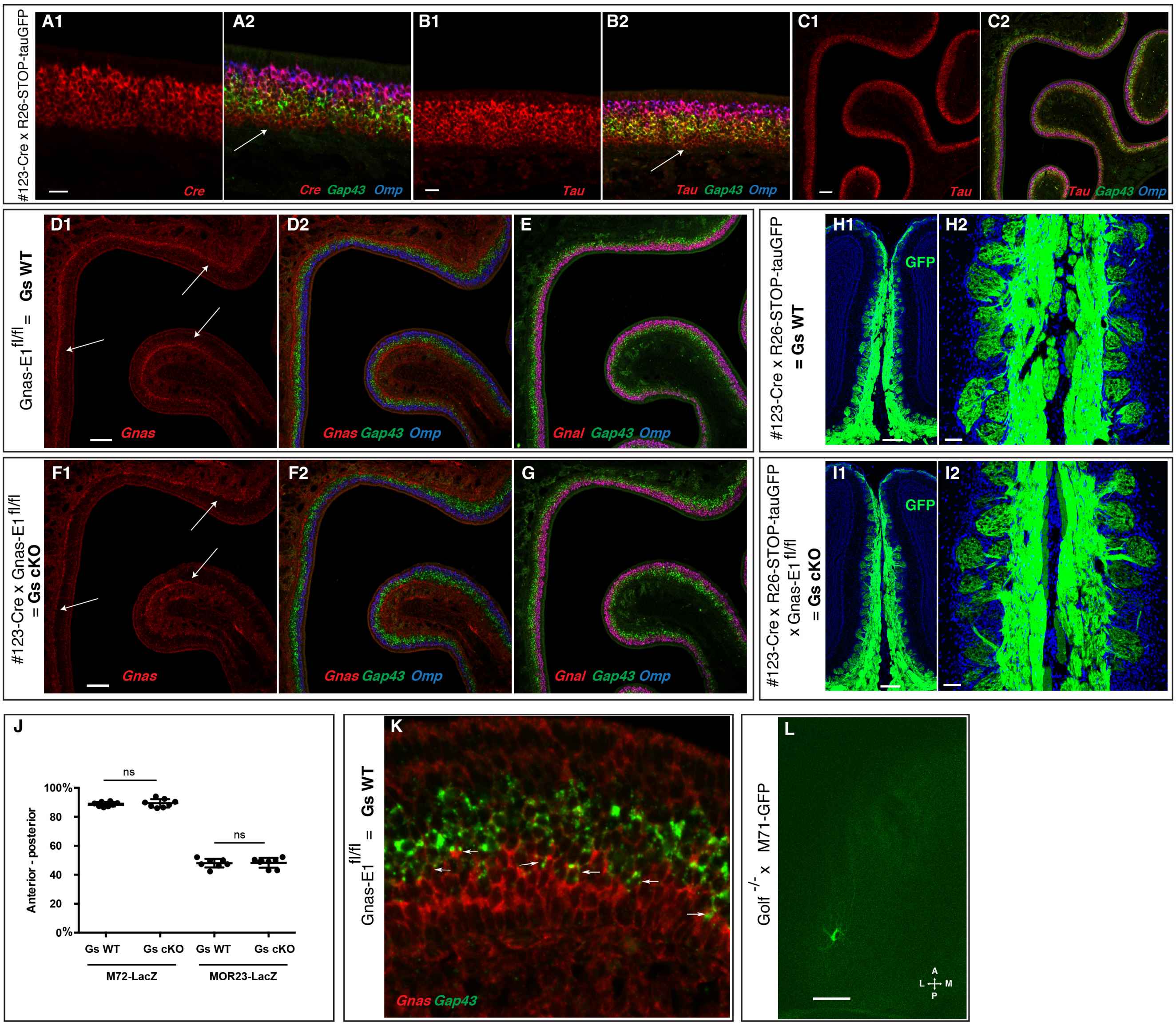

#123-Cre x R26-STOP-tauGFP
A1
Cre
A2
Cre Gap43 Omp
B1
Tau
B2
Tau Gap43 Omp
C1
Tau
C2
Tau Gap43 Omp
Gnas-E1 fl/fl = Gs WT
D1
Gnas
D2
Gnas Gap43 Omp
E
Gnal Gap43 Omp
#123-Cre x R26-STOP-tauGFP = Gs WT
H1
GFP
H2
#123-Cre x Gnas-E1 fl/fl = Gs cKO
F1
Gnas
F2
Gnas Gap43 Omp
G
Gnal Gap43 Omp
#123-Cre x R26-STOP-tauGFP x Gnas-E1 fl/fl = Gs cKO
I1
GFP
I2
J
Anterior - posterior
100%
80
60
40
20
0%
ns
ns
Gs WT
Gs cKO
Gs WT
Gs cKO
M72-LacZ
MOR23-LacZ
K
Gnas-E1 fl/fl = Gs WT
Gnas Gap43
L
Golf -/- x M71-GFP
A
L
M
P

Figure S2. OSNs expressing G-protein coupling M71 mutant ORs are gradually eliminated from the epithelium and their axons are rarely observed on the bulb.

Medial view of X-gal-stained wholemounts of (A) M71-LacZ (3wo) and (B) M71(RDY)-lacZ (PD10) mice. (C) Higher-magnification of X-gal-stained M71(RDY) MOE (PD10), shows that OSNs poorly project axons, which appear short and stunted. (D) Number of X-gal+ OSNs on wholemounts of M71(RDY)-LacZ turbinates at indicated postnatal days (mean ± SEM, n=6-11 for each time point). (E) IHC was performed against: β-galactosidase for counting M71 and M71(RDY) OSNs; GFP for counting M71(RDY)-caGs OSNs; and DsRed for counting MOR23(RDY)→M71-caGs OSNs on coronal sections at PD21. Every 10th section was collected from anterior to posterior and 49 sections were counted per mouse. Data are mean ± SEM (n=3). One-way ANOVA and Newman–Keuls post test (D, E). $*p < 0.05$, $**p < 0.01$, $***p < 0.001$. Scale bars, 500 μm.

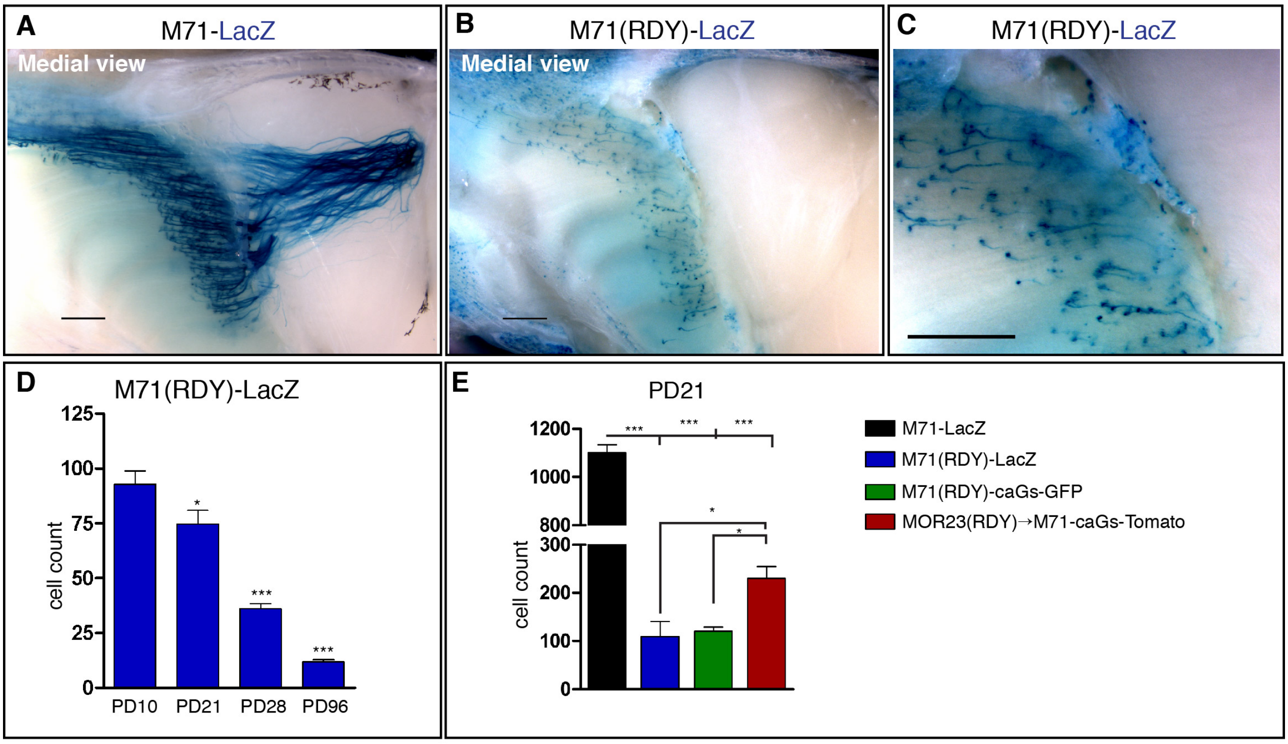

Figure S3. O/E2-M71-GFP mice ubiquitously express *M71*.

(A) Two-colour ISH on coronal sections of the MOE from WT mice with probes against *M71* (red-A1) plus *O/E2* (green-A2). (B) Three-colour ISH against *Gap43* (green), *Omp* (blue) and the sustentacular marker *Cbr2* (red), in the MOE of WT mice. (C) Two-colour ISH on coronal sections of the MOE from heterozygous O/E2-M71-GFP mice with probes against *M71* (red-C1) plus *O/E2* (green-C2). Some red labelled neurons in the dorsal epithelium appeared more intense suggesting that endogenous M71/M72 ORs are expressed at a slightly higher level than those from the O/E2 promoter. (D) Three-colour ISH against *Gap43* (green), *Omp* (blue) and the sustentacular marker *Cbr2* (red), in the MOE of heterozygous O/E2-M71-GFP mice. Scale bars, 100 μm (A2, C2), 50 μm (B,D).

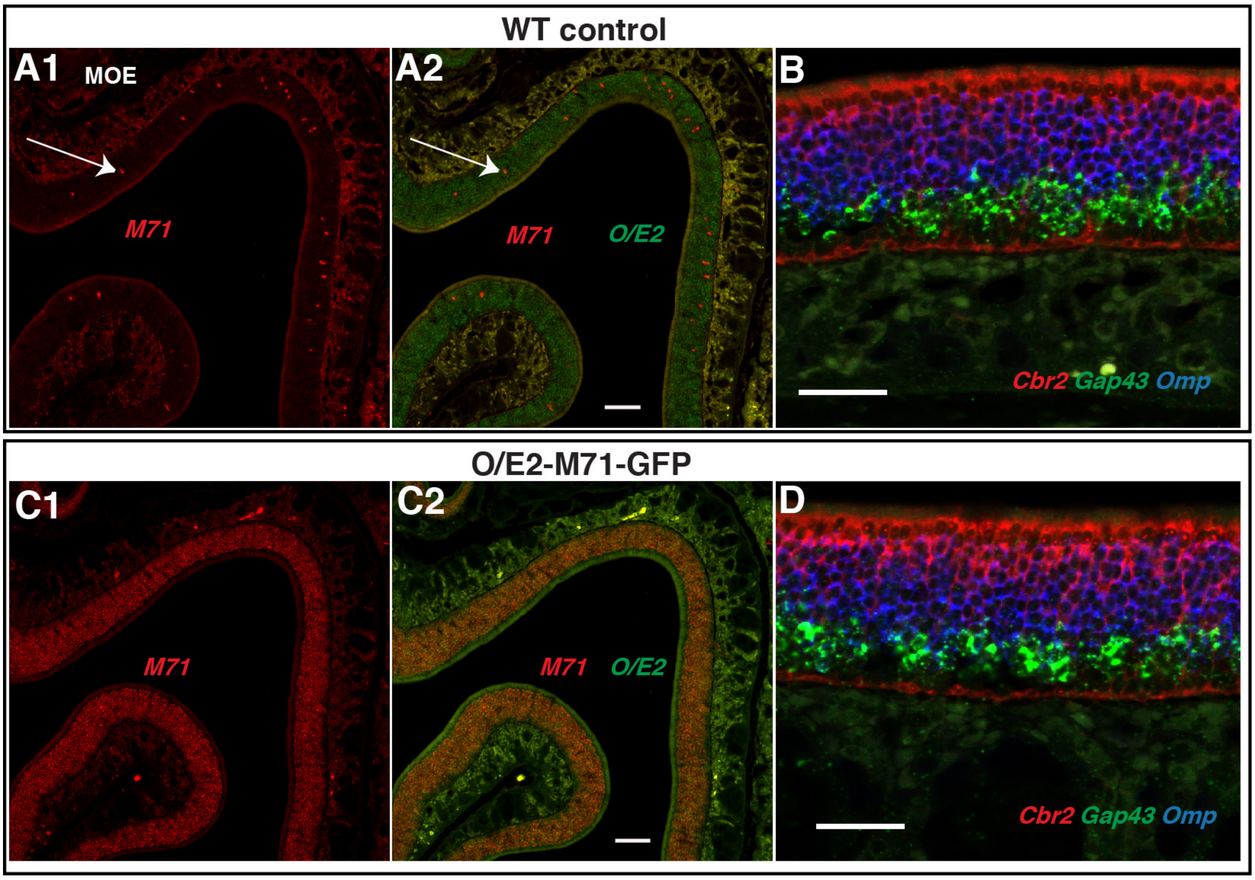

Figure S4. O/E2-M71 expression affects axonal projections of OSNs expressing endogenous ORs.

(A) In the P-LacZ transgenic mice, wholemount X-gal staining of the dorsal bulb labels axons expressing class II ORs, while the unlabelled butterfly-shaped pattern corresponds to the class I and TAAR domains [48]. Interestingly, in O/E2-M71-GFP mutants (right, O/E2 +/-), this butterfly pattern was disrupted, as LacZ+ axons now traversed through the dorsal-medial regions. (B-C) Wholemount X-gal staining of (B) MOR23-lacZ and (C) M71-lacZ OSNs in O/E2-M71-GFP heterozygous mice. Three representative examples are shown for two ORs, showing how O/E2-M71 expression alters the axonal projections of OSNs. Scale bars, 500 μm.

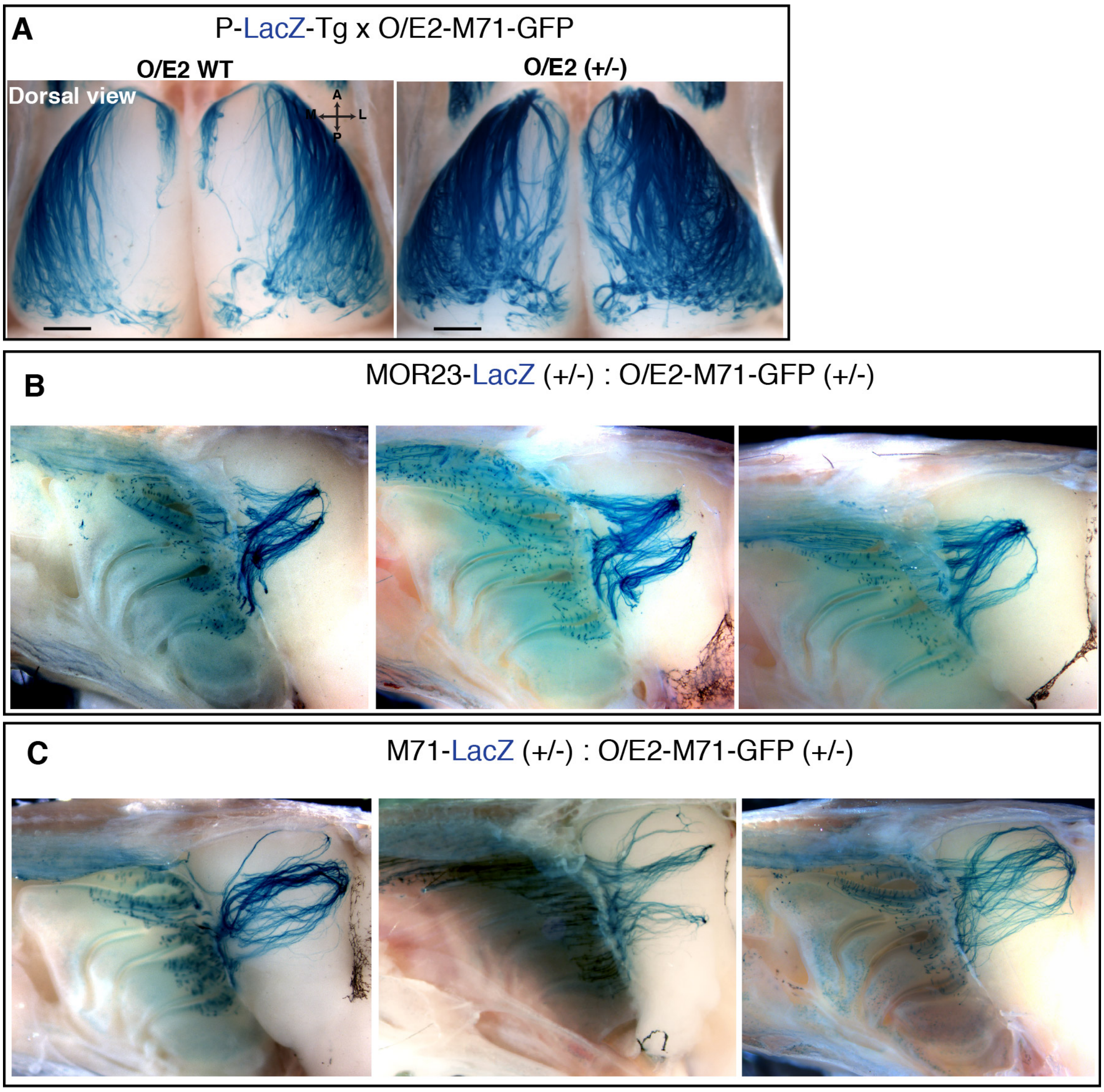

Figure S5. Previously described mouse strains with gene-targeted OR mutations and their abbreviations that were used throughout the study.

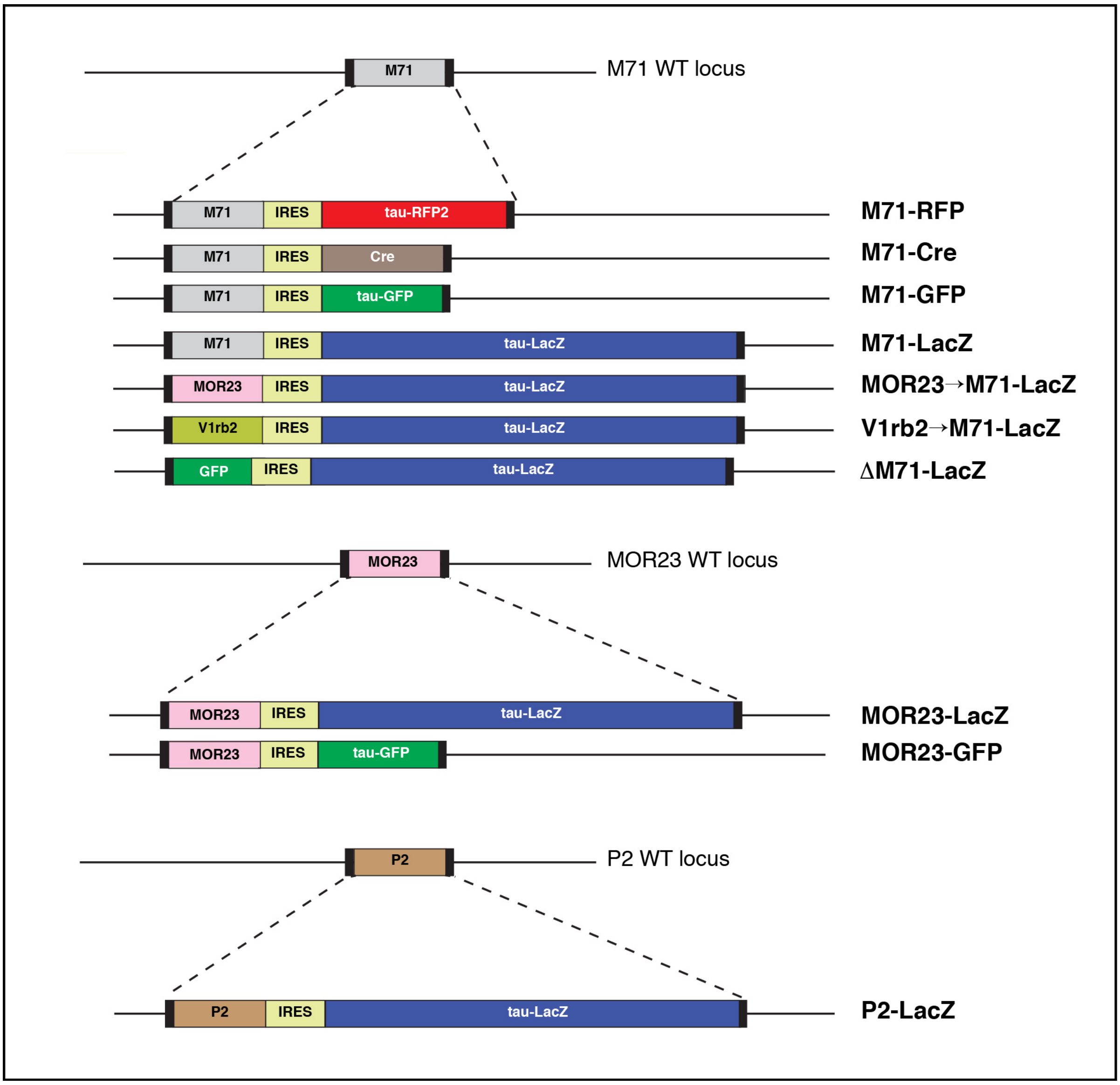

Table S1. List of ISH Probes that were used.

Riboprobe	Sequence	Reference
Gnas	nt 408–1512 from NM_001077510.2	(Omura et al., 2014)
Gnal	nt 1–1106 from NM_ 010307	(Omura et al., 2014)
Omp	nt 820-2891 from U01213	(Ishii et al., 2004)
Gap43	nt 147-860 from NM_008083	(Ishii et al., 2004)
Cbr2	nt 5-466 from BC010758	(Ishii et al., 2004)
Adcy3	nt 2853-3559 from NM_001159536	(Hirota et al., 2007)
Cre	nt 485-1516 from X03453	(Li et al., 2004)
mCherry (for tdTomato)	nt 21-512 from AY678264	(Omura et al., 2014)
GFP	nt 97-816 from U76561.1	(Li et al., 2004)
*tau (*bovine)	nt 102-621 from NM_174106.2	(Ishii and Mombaerts, 2008)
lacZ	nt 73035-73522 from U73857.1	
O/E2	nt 2353-3392 from NM_001113415.1	
M71	nt 144-1013 from NM_207664.2	(Fuss et al., 2007)
Olfr3 (class II mix)	nt 17-871 from NM_206903.1	(Omura et al., 2014)
Olfr62 (class II mix)	nt 167-883 from NM_146315.2	(Omura et al., 2014)
Olfr54 (class II mix)	nt 1-942 from NM_010997.1	(Omura et al., 2014)
Olfr749 (class II mix)	nt 73-984 from NM_020288.2	(Fuss et al., 2007)

Table S2. List of NanoString probes that were used.

Gene	Accession number	Target region
Omp (ref gene 1)	NM_011010.2	1148-1247
Gnal (ref gene 2)	NM_177137.4	3129-3228
Adcy3 (ref gene 3)	NM_001159537.1	3377-3476
Ano2 (ref gene 4)	NM_153589.2	3346-3445
Cnga2 (ref gene 5)	NM_007724.2	54-153
Olfr1015	NM_146571.2	761-860
Olfr1156	NM_146817.2	397-496
Olfr124	NM_147062.2	536-635
Olfr1301	NM_146887.1	351-450
Olfr15	NM_008762.2	31-130
Olfr1507	NM_001170918.1	731-830
Olfr1508	NM_020513.2	783-882
Olfr1509	NM_020514.2	371-470
Olfr1511	NM_146271.2	196-295
Olfr16	NM_008763.2	401-500
Olfr160	NM_030553.2	385-484
Olfr166	NM_147068.1	39-138
Olfr17	NM_020598.1	754-853
Olfr2	NM_010983.1	771-870
Olfr309	NM_001011866.1	401-500
Olfr6	NM_206897.1	767-866
Olfr713	NM_147034.1	837-936
Olfr73	NM_054090.1	406-505
Olfr78	NM_001168503.1	661-760
Kirrel2	NM_172898.3	2763-2862
Kirrel3	NM_001190914.1	113-212
Epha5	NM_007937.3	2006-2105
Efna5	NM_207654.2	1131-1230
Sema3f	NM_011349.3	2576-2675

Sema3a	NM_009152.3	3116-3215
Nrp2	NM_001077403.1	611-710
Nrp1	NM_008737.2	3596-3695
Plxna1	NM_008881.2	8201-8300
Plxna3	NM_008883.2	6153-6252
